# The localization, origin, and impact of platelets in the tumor microenvironment are tumor type-dependent

**DOI:** 10.1186/s13046-024-03001-2

**Published:** 2024-03-16

**Authors:** Ophélie Le Chapelain, Soumaya Jadoui, Angèle Gros, Samir Barbaria, Keltouma Benmeziane, Véronique Ollivier, Sébastien Dupont, Mialitiana Solo Nomenjanahary, Sabrina Mavouna, Jasmina Rogozarski, Marie-Anne Mawhin, Giuseppina Caligiuri, Sandrine Delbosc, Françoise Porteu, Bernhard Nieswandt, Pierre H Mangin, Yacine Boulaftali, Benoit Ho-Tin-Noé

**Affiliations:** 1 Faculté de Pharmacie de Paris, Université Paris Cité, Inserm UMR-S 1144 -Optimisation Thérapeutique en Neuropsychopharmacologie, 4 avenue de l’Observatoire, Paris, 75006 France; 2 Université Paris Cité, INSERM UMR 1148, LVTS, Paris, F-75018 France; 3 Institut Gustave Roussy, INSERM UMR 1287, Villejuif, France; 4 Institute of Experimental Biomedicine I, University Hospital Würzburg and Rudolf Virchow Center for Integrative and Translational Bioimaging, University of Würzburg, Würzburg, Germany; 5 Université de Strasbourg, Institut National de la Santé et de la Recherche Médicale, Etablissement Français du Sang Grand-Est, Unité Mixte de Recherche-S1255, Fédération de Médecine Translationnelle de Strasbourg, Strasbourg, F-67065 France

**Keywords:** Platelets, Solid tumors, Tumor microenvironment, Vascular integrity, Thrombocytopenia

## Abstract

**Background:**

How platelets interact with and influence the tumor microenvironment (TME) remains poorly characterized.

**Methods:**

We compared the presence and participation of platelets in the TME of two tumors characterized by highly different TME, PyMT AT-3 mammary tumors and B16F1 melanoma.

**Results:**

We show that whereas firmly adherent platelets continuously line tumor vessels of both AT-3 and B16F1 tumors, abundant extravascular stromal clusters of platelets from thrombopoietin-independent origin were present only in AT-3 mammary tumors. We further show that platelets influence the angiogenic and inflammatory profiles of AT-3 and B16F1 tumors, though with very different outcomes according to tumor type. Whereas thrombocytopenia increased bleeding in both tumor types, it further caused severe endothelial degeneration associated with massive vascular leakage, tumor swelling, and increased infiltration of cytotoxic cells, only in AT-3 tumors.

**Conclusions:**

These results indicate that while platelets are integral components of solid tumors, their localization and origin in the TME, as well as their impact on its shaping, are tumor type-dependent.

**Supplementary Information:**

The online version contains supplementary material available at 10.1186/s13046-024-03001-2.

## Introduction

The possible contribution of platelets to cancer progression has been mainly considered through their ability to promote experimental metastasis. The pro-metastatic effect of platelets was initially highlighted in the early study by Gasic et al. in which it was shown that thrombocytopenia reduced lung metastasis from intraperitoneal ascites tumor cells in mice [1]. Platelets have since been shown to support experimental metastasis by multiple, non-exclusive mechanisms. Those include the stimulation of epithelial-mesenchymal transition (EMT) [2], the protection of circulating tumor cells from immune cells [3], or the facilitation of circulating tumor cell arrest and extravasation at distant sites [4–6].

There is evidence that the contribution of platelets to the pathophysiology of cancers is not limited to the dissemination and thrombotic complications of cancers. Several studies have reported an impact of platelets on primary tumor growth in various mouse models of solid cancers [7–10]. Yet, depending on the experimental design (i.e. tumor model, type of anti-platelet strategy), the impact of platelets on tumor growth could be either positive [8, 11, 12] or negative [13], precluding definitive conclusions about the interest of targeting platelets in cancers. In line with this, platelets and their secreted factors can exert opposite effects on tumor cell proliferation and survival depending on the tumor cell line [13–15]. Besides direct regulation of tumor cell proliferation, promotion of angiogenesis and stabilization of tumor vessels are among the possible mechanisms by which platelets may support tumor growth indirectly. Indeed, platelets can stimulate angiogenesis [16, 17], a process central to tumor progression, and they have been shown to continuously prevent harmful (to the tumor) intratumor hemorrhage by repairing immune cell-induced breaches in the tumor vasculature [11, 18, 19]. In addition to the tumor vasculature, platelets may also influence the tumor inflammatory and immune microenvironment [20, 21]. Despite these data indicating that platelets regulate solid tumor development, the presence, localization, and participation of platelets in the microenvironment of solid tumors remain poorly characterized.

In order to determine the participation of platelets in the TME of solid tumors, we investigated the presence and localization of platelets in the microenvironment of two different mouse models of orthotopically-implanted tumors, B16F1 melanomas and AT-3 MMTV-PyMT mammary tumors. We further compared the impact of severe chronic thrombocytopenia on these tumors’ development and TME shaping, with results highlighting that platelets exert tumor-type dependent effects.

## Materials and methods

### Mice

c-mpl^−/−^ mice [22] and their control littermates on a C57BL/6 background were bred in our animal facility. GPVI^−/−^ mice [23] and their control littermates on a C57BL/6 background were bred in our animal facility. All procedures were approved by the local animal ethics committee registered with the French Ministry of Research (APAFIS project authorization#31821-2021052715257618).

### Tumor models

The mouse B16F1 melanoma cell line characterized by a low metastatic potential [24], was purchased from ATCC. The mouse AT-3 mammary cell line, derived from a mammary carcinoma of MMTV-PyMT transgenic mice on a C57BL/6 background [25–29]was a kind gift from Scott Abrams (Roswell Park Comprehensive Cancer Center, Buffalo, NY, USA). B16F1 and AT-3 cells were cultured in Dulbecco’s modified Eagle’s medium (4.5 g/L glucose for B16F1 cells, 1 g/L glucose for AT-3 cells) supplemented with 10% FBS, 1% glutamine, and 1% penicillin-streptomycin. B16F1 cells (1 × 10^6^) were injected subcutaneously into the dorsal skin of 10- to 15-week-old male mice. AT-3 cells (2 × 10^5^) were injected bilaterally into the inguinal mammary fat pads of 10- to 15-week-old virgin female mice. B16F1 and AT-3 tumors were allowed to grow for 2 and 3 weeks, respectively. In a subset of experiments in female mice, quantities, length of experiment, and injection sites were inverted between B16F1 and AT-3 cells.

### Induction of thrombocytopenia

Profound thrombocytopenia was induced on the day of tumor cell implantation and maintained over the course of the experiments by intravenous injection of a platelet-depleting rat polyclonal antibody (R300, 0.5 µg/g mouse, Emfret Analytics) in c-mpl^-/-^ mice, every 5 days. This protocol allowed to further decrease the platelet count from 189 ± 61 × 10^3^ platelets/µL for untreated c-mpl^-/-^ mice to 98.05 ± 67 × 10^3^ platelet/µL after R300 treatment (mean ± SD, *p* < 0.0005). Control c-mpl^+/+^ mice were injected with nonimmune rat IgG (C301, Emfret Analytics) and had a mean platelet count of 1313.71 ± 277 × 10^3^ platelet/µL. For these experiment, B16F1 and AT-3 tumors were allowed to grow for 2 weeks.

### Histology and immunofluroscence analysis

Detailed protocols for immunostainings are given in the Supplementary data.

### Intravital microscopy

Intravital observation of fluorescently labeled platelets in B16F1 and AT-3 cells was performed through skinfold chambers [30], as described in the Supplementary data.

### Tumor blood perfusion and vascular permeability

For assessment of tumor blood perfusion, fluorescein isothiocyanate dextran (FITC-dextran, 150 kDa, 130 µg/g mouse Sigma-Aldrich) was injected through the retro-orbital venous sinus, 5 min before euthanasia.

For assessment of vascular permeability, a subset of tumor-bearing mice was euthanized 1 h after FITC-dextran injection (2,000 kDa, 65 µg/g mouse).

### Determination of tumor hemoglobin content and ELISAs

Hemoglobin content was determined by measurement of heme concentration in tumor extracts as described previously [31]. Intratumor CD8, granzyme B, and myeloperoxidase (MPO) levels were quantified using commercial ELISA kits. Plasma PECAM-1 was measured using a microsphere-based flow cytometric assay described in the Supplementary data.

### Proteome Profiler array

Tumor cytokine and angiogenic profiles were determined using the Mouse XL Cytokine and Angiogenesis Proteome Profiler Array kits (R&D Systems).

### Statistical analysis

Data are presented as mean ± SEM and were compared using the Wilcoxon signed-rank or Mann-Whitney test, when appropriate. Correlations were determined using the Spearman rank test. Statistical significance was set at 5%.

## Results

### Comparison of the immune and vascular microenvironments of B16F1 and PYMT AT-3 tumors

**Fig. 1:**
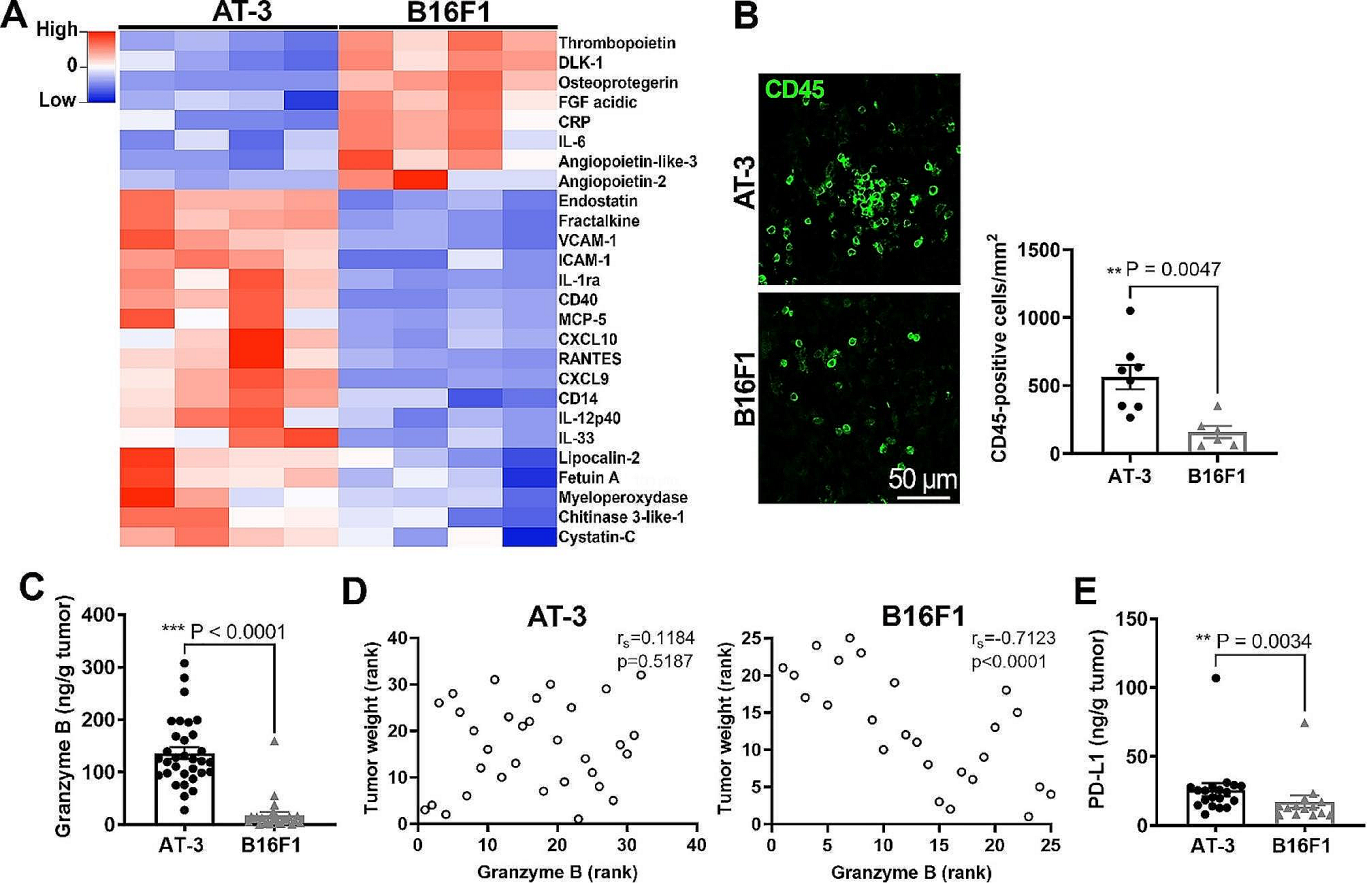
Inflammatory profiles of AT-3 mammary tumors and B16F1 melanoma. AT-3 and B16F1 tumors were grown in C57BL/6 mice for 21 days and 14 days, respectively. **(A)** Comparative heatmap showing cytokines and chemokines expressed in AT-3 and B16F1 tumors using Proteome Profiler array (*n* = 4 tumors, *p* < 0.05). **(B)** Representative images and quantification of CD45 staining in AT-3 and B16F1 tumors. *n* = 8 AT-3 and 6 B16F1 tumors, each dot represents the mean value calculated from 5 random images taken with a 10x objective per tumor. **(C)** Comparison of granzyme B levels in AT-3 and B16F1 tumor extracts. (*n* = 32 AT-3 and 25 B16F1 tumors). **(D)** Scatter plot of Spearman’s rank correlation between tumor weight and granzyme B in AT-3 (*n* = 32) and B16F1 tumors (*n* = 25). **(E)** Comparison of PD-L1 levels in AT-3 (*n* = 19) and B16F1 tumors (*n* = 14)

**Fig. 2:**
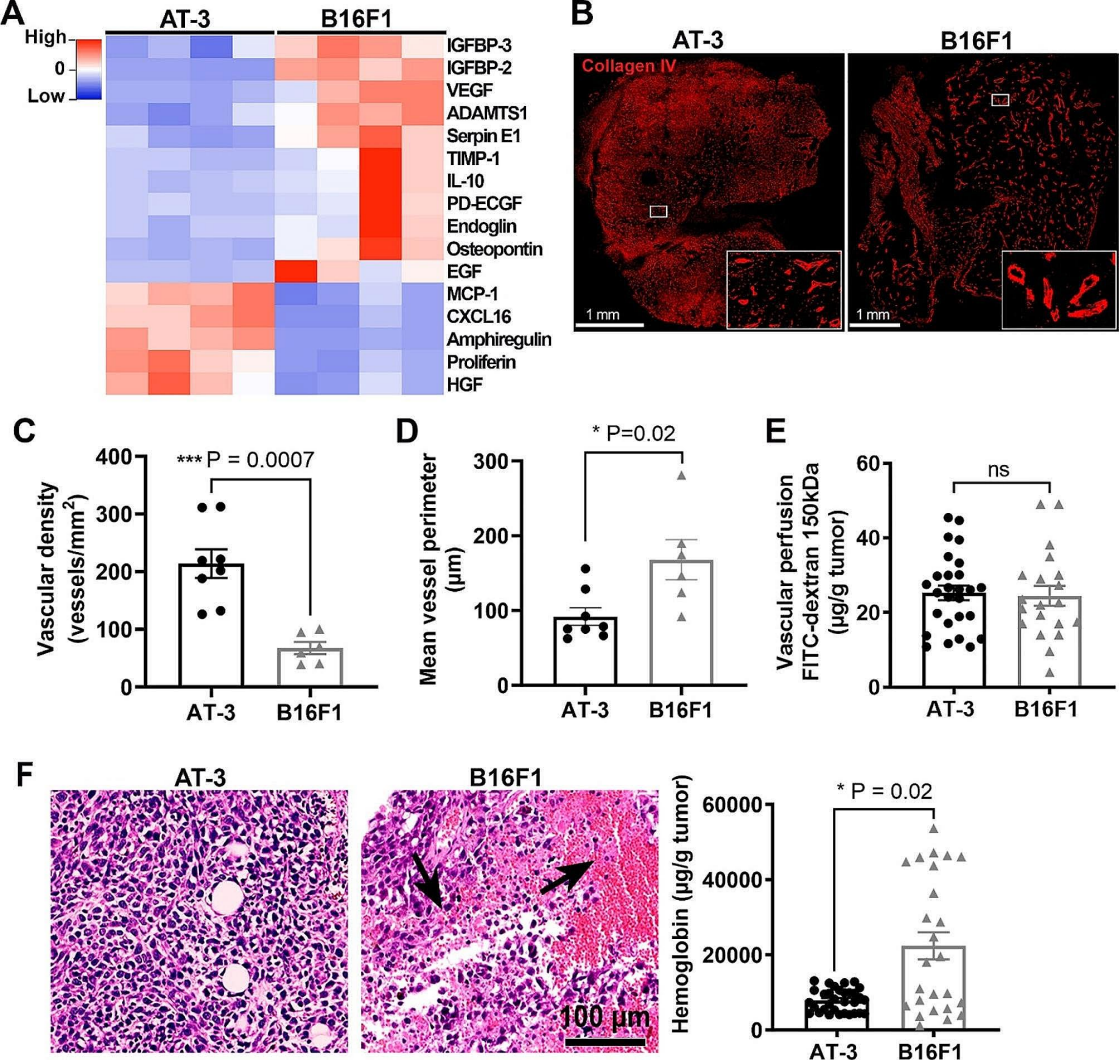
Angiogenic profiles of AT-3 mammary tumors and B16F1 melanoma. **(A)** Comparative heatmap of angiogenic factors expressed in AT-3 and B16F1 tumor extracts using Proteome Profiler array (*n* = 4 tumors, *p* < 0.05). **(B)** Representative images of collagen IV staining in AT-3 and B16F1 tumors. **(C)** Quantification of blood vessels identified by collagen IV staining in AT-3 and B16F1 tumors. *n* = 8 AT-3 and 6 B16F1 tumors, each dot represents the mean value calculated from 5 random images taken with a 10x objective per tumor. **(D)** Quantification of mean vessel perimeter in AT-3 and B16F1 tumors. *n* = 8 AT-3 and 6 B16F1 tumors; each dot represents the mean value calculated from 10 random images taken with a 10x objective per tumor. **(E)** Tumor vascular perfusion as assessed by measurement of intratumor FITC-dextran content of B16F1 and AT-3 tumors explanted 5 min after intravenous injection of FITC-dextran, 150 kDa. (*n* = 27 AT-3 and 20 B16F1 tumors). **(F)** H&E staining of both tumor types showing tumor bleeding only in B16F1 tumors. Arrows indicate intratumor hemorrhage. Comparison of intratumor hemoglobin content between AT-3 (*n* = 32) and B16F1 tumors (*n* = 25). ns, non-significant

Before evaluating the platelet contribution to the TME, we first characterized and compared the immune and vascular microenvironments of B16F1 melanoma and AT-3 mammary tumors. Semiquantitative proteome array analysis of cytokines in AT-3 and B16F1 tumors showed drastic differences between these two types of solid tumors. In particular, as compared to B16F1 melanoma, AT-3 tumors were enriched in mediators of leukocyte infiltration, including chemokines like RANTES, MCP-1 and − 5, fractalkine, CXCL9, 10 and 16, as well as endothelial adhesion molecules like ICAM-1 and VCAM-1 (Figs. 1A and 2A). Consistent with these differences, markers of neutrophil infiltration (myeloperoxidase (MPO) and lipocalin-2) were also more elevated in tumor extracts from AT-3 tumors (Fig. 1A, Supplementary Figs. 1–2). In addition, immunostaining for the pan leukocyte antigen CD45 showed that CD45-positive cells were more abundant in the stroma of AT-3 tumors than in that of B16F1 tumors (Fig. 1B). With respect to circulating leukocytes, the development of both types of tumors led to a significant increase in blood monocyte and neutrophil counts (Supplementary Fig. 3A-B).

In order to compare the propensity of B16F1 and AT-3 of tumors to elicit antitumor immunity, we estimated their cytotoxic cell content by quantitative measurement of the NK and CD8 marker granzyme B (Fig. 1C). Granzyme B content was significantly and drastically higher in AT-3 tumors than in B16F1 tumors (Fig. 1C), a result consistent with their increased content in the CXCR3 ligands [32], CXCL9 and 10 (Fig. 1A). Strikingly, whereas tumor weight was negatively correlated with tumor granzyme B content in B16F1 tumors (Spearman’s rank correlation coefficient, rho = -0.7, *p* < 0.0001, *n* = 25), there was no association between tumor weight and granzyme B content in AT-3 tumors (Spearman’s rank correlation coefficient, rho = 0.118, *p* = 0.518, *n* = 32) (Fig. 1D), which were characterized by higher levels of programmed death-ligand 1 (PD-L1) (Fig. 1E).

Proteome array analysis of angiogenesis-related factors in tumor extracts also showed marked differences between AT-3 and B16F1 tumors (Fig. 2A), suggesting possible quantitative or qualitative differences in vascularization. Differences in the vascular phenotype of the two types of tumors were confirmed by immunohistological analysis. AT-3 tumors displayed a higher vascular density and reduced mean vessel perimeter compared to B16F1 tumors (Fig. 2B-D). Despite these differences, tumor vascular perfusion, as evaluated by intravenous injection of FITC-dextran just before sacrifice, was similar between AT-3 and B16F1 tumors (Fig. 2E). In contrast to AT3-tumors, B16F1 tumors frequently presented intratumor hemorrhage (Fig. 2F). The increased hemorrhagic trend of B16F1 tumors was confirmed by their increased hemoglobin content compared to AT-3 tumors (Fig. 2F).

Altogether these results show that B16F1 and AT-3 tumors have drastically different immune and vascular microenvironments. Notably, the decreased content in CD45-positive cells and reduced vessel density of B16F1 tumors, as compared to mammary AT-3 tumors, was maintained when B16F1 tumors were grown for 21 days in mammary fat pads of female mice, thus indicating that the main distinguishable features between these tumor types were irrespective of female sex, the implantation site, or length of experiment (Supplementary Fig. 4).

### Platelets are constitutive components of the microenvironment of both AT-3 and B16F1 tumors

**Fig. 3:**
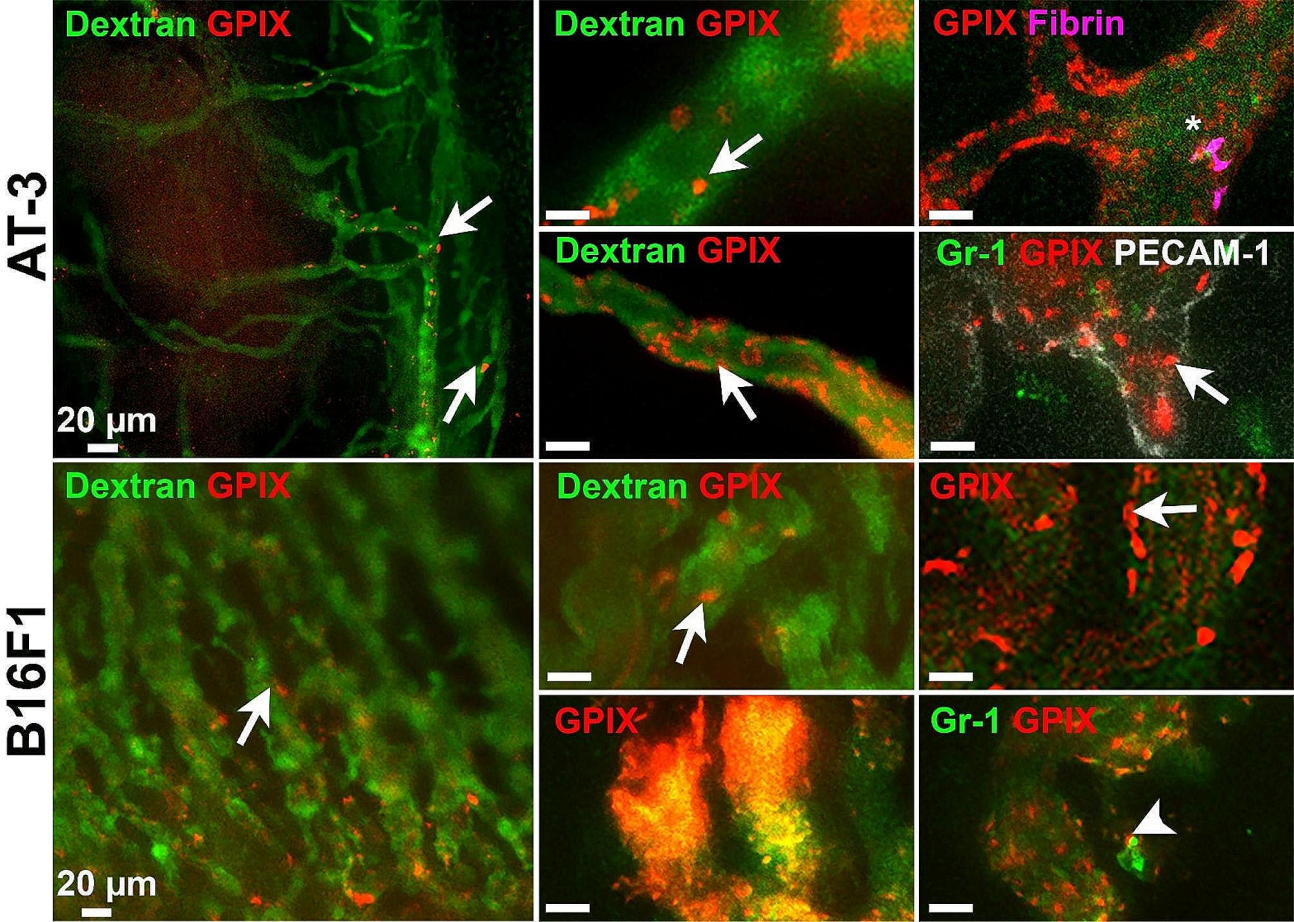
Continuous presence of intravascular and firmly adherent platelets in AT-3 mammary tumors and B16F1 melanoma. AT-3 or B16F1 cells were injected subcutaneously in the dorsal skin and allowed to grow for 5 to 7 days before surgical implantation of a dorsal skinfold chamber. Representative intravital images of the microcirculation of B16F1 and AT-3 tumors in mice injected with FITC-dextran and fluorescent antibodies to GPIX, fibrin, Gr-1, or PECAM-1, as indicated. White arrow indicates individual platelets adhering firmly to tumor vessels. The asterisk indicates a platelet-fibrin deposit. The white arrowhead shows a platelet-neutrophil complex adhering to tumor vessels. Bar = 10μm

In order to characterize platelet-tumor interactions, we first investigated whether the development of B16F1 and AT-3 tumors was associated with an increase in platelet count, as frequently reported in clinical studies. Blood cell count analysis showed that, in both types of tumors, an increase in circulating platelet levels occurred as the tumor grew (Supplementary Fig. 3C). With respect to the presence of platelets at the tumor site, intravital microscopy analysis revealed the continuous presence of numerous firmly adherent intravascular platelets lining the vasculature, in both tumor types (Supplementary Movie 1–7 and Fig. 3). In most instances, firm adhesion of platelets to tumor vessels lasted for over several minutes (Supplementary Movie 1–7). This was in stark contrast with the rare, transient, and short interactions seen between platelets and healthy vessels of non-tumor-bearing mice (Supplementary Movie 8). Firmly adherent platelets in tumor vessels and angiogenic sprouts were found mainly under the form of individual, non-aggregated platelets (Supplementary Movie 2–4,6,7). Occasionally, adherent platelets could be seen associated with neutrophils and fibrin deposits (Supplementary Movie 9,10 and Fig. 3). Besides individual platelets, occlusive microthrombi were frequently observed in the vasculature of B16F1 tumors (Supplementary Movie 10), but much more scarcely in AT-3 tumors. Notably, no transmigration of isolated or neutrophil-associated platelets outside the tumor vasculature was observed over up to 3 h of intravital imaging.

**Fig. 4:**
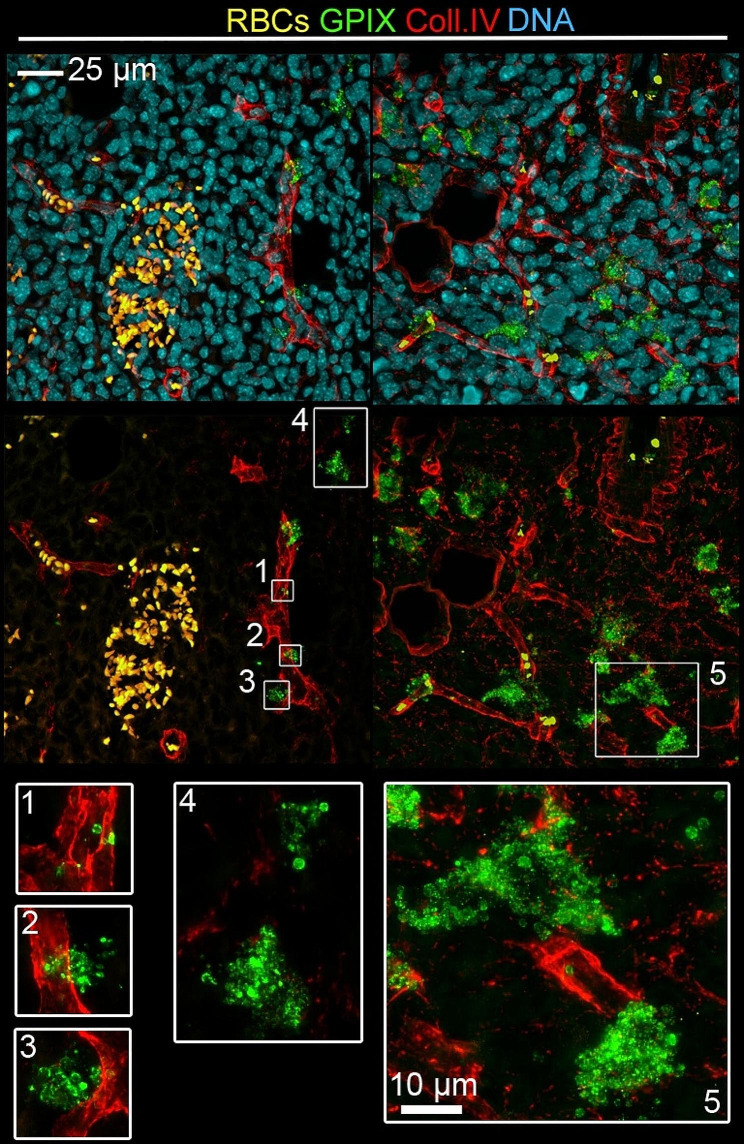
Platelets are constitutive components of the microenvironment of AT-3 mammary tumors. Representative images of GPIX and collagen IV staining in AT-3 tumors showing the presence of platelets with different types of interactions with tumors: intravascular platelets (1), perivascular platelets (2, 3), abundance of extravascular platelet clusters not associated with hemorrhagic areas (4, 5). White boxes show higher magnification of GPIX-positive platelets

**Fig. 5:**
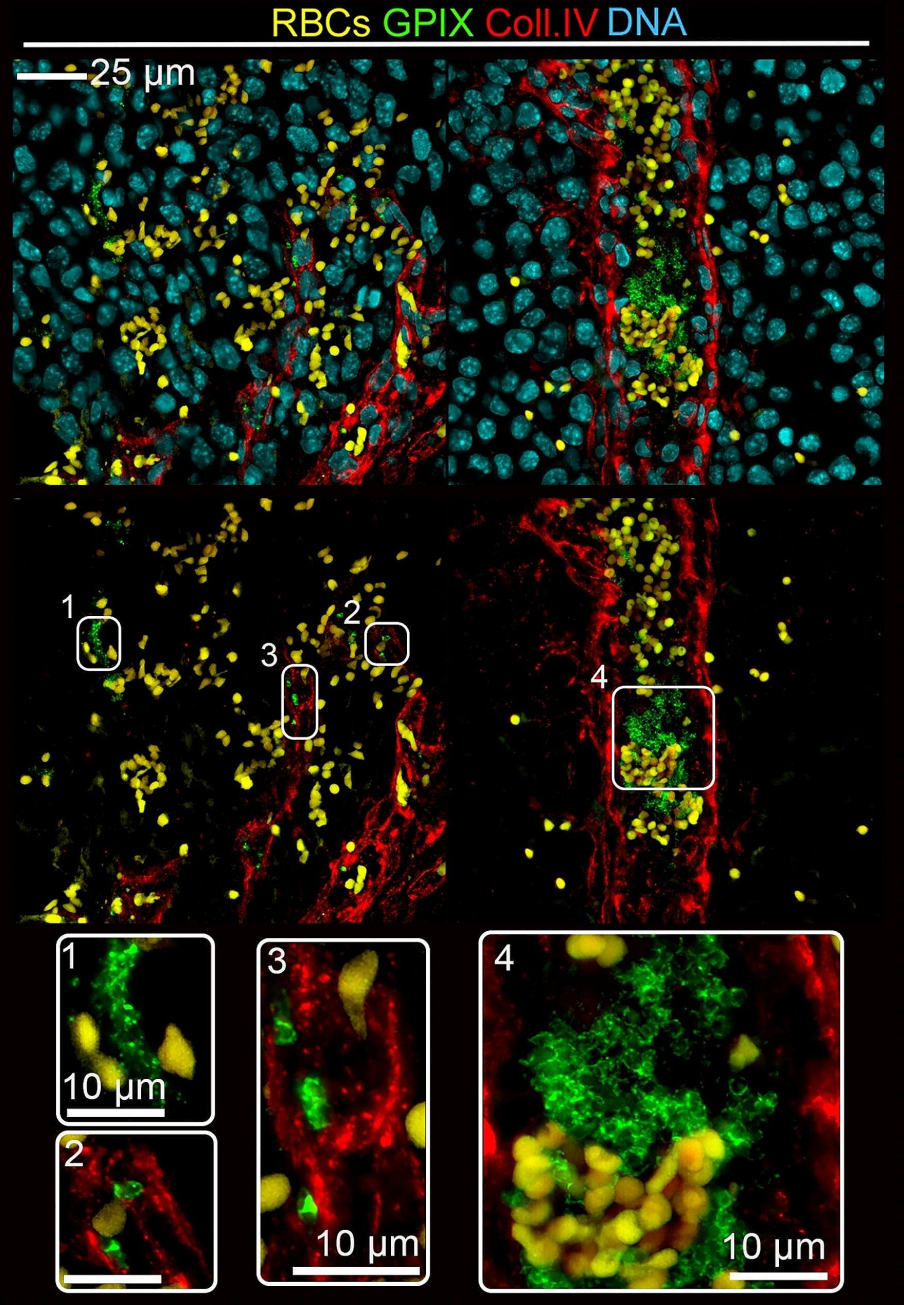
Platelets are constitutive components of the microenvironment of B16F1 melanoma. Representative images of GPIX and collagen IV staining in B16F1 tumors showing the presence of platelets with different types of interactions with tumors: isolated intravascular platelets (2,3) or intravascular platelet aggregates (4) as well as the presence of extravascular platelets systematically in association with red blood cells (1). White boxes show higher magnification of GPIX-positive platelets

The presence of platelets in B16F1 and AT-3 tumors was further investigated and confirmed by immunostaining of tumor sections. In addition to platelets lining the inner layer of tumor vessels, perivascular platelets were found in AT-3 tumors (Fig. 4) and B16F1 tumors. In most instances, perivascular platelets were found irrespective of the presence of red blood cells and leukocytes (Fig. 4). In agreement with intravital imaging, platelets in B16F1 tumors were also routinely present as part of intravascular thrombi (Fig. 5). Strikingly, as compared to B16F1 tumors, AT-3 tumors were characterized by the additional presence of abundant clusters of extravascular platelets. Noteworthy, abundant extravascular platelet clusters were still present in AT-3 tumors grown in the back skin, and remained absent of B16F1 tumors grown in mammary fat pads (Supplementary Fig. 5), indicating that this latter platelet localization was mainly dependent on the tumor cell line rather than on the tumor host organ. Like perivascular platelets, these extravascular clusters were found irrespective of signs of bleeding and leukocyte infiltration, as indicated by the absence of associated red blood cells (Fig. 4) or CD45-positive cells (Supplementary Fig. 6). In contrast to intra- and perivascular platelets, which could be detected using antibodies to either platelet surface glycoproteins (GPVI, GPIX and GPIb) or platelet alpha granule markers (PF4 and vWF), stromal extravascular platelets in AT-3 tumors expressed only a subset of platelet markers. Indeed, whereas stromal extravascular platelets in AT-3 tumors were positive for the platelet membrane marker GPIX (Fig. 4), as well as the platelet granule markers PF4 (Supplementary Fig. 7A) and vWF (Supplementary Fig. 7B), they were negative for GPVI and GPIb. Notably, extravascular platelets were absent from control mammary glands (Supplementary Fig. 8). Taken together, these results indicate that platelets are constitutive components of the tumor microenvironment, with a sustained intratumor presence in intra and perivascular spaces, which can extend to the formation of extravascular clusters, depending on the tumor type.

**Fig. 6:**
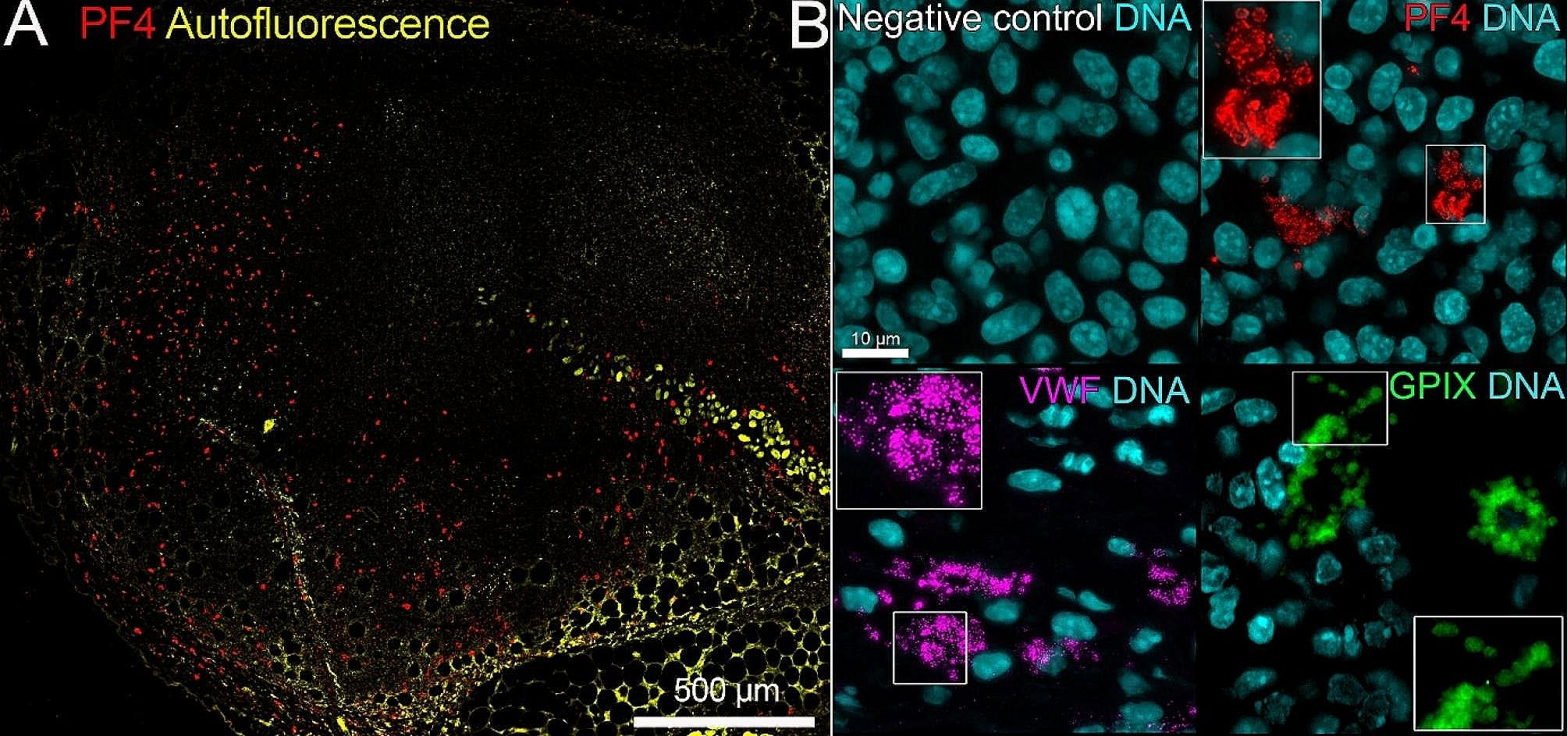
Extravascular platelet clusters in AT-3 tumors from chronic and severe thrombocytopenic mice. (A) Representative image of immunostaining for platelet factor 4 (PF4) in mammary AT-3 tumors from mice with chronic severe thrombocytopenia (c-mpl−/− mice treated with R300 antibody). Note the abundance of platelet clusters at the tumor periphery. (B) Immunostaining for platelets (PF4, VWF, GPIX) showing the presence of extravascular platelet clusters in AT-3 tumors from mice with chronic severe thrombocytopenia. White boxes show higher magnification views of the squared areas

### Thrombocytopenia has tumor-type specific consequences on tumor vascular integrity

**Fig. 7:**
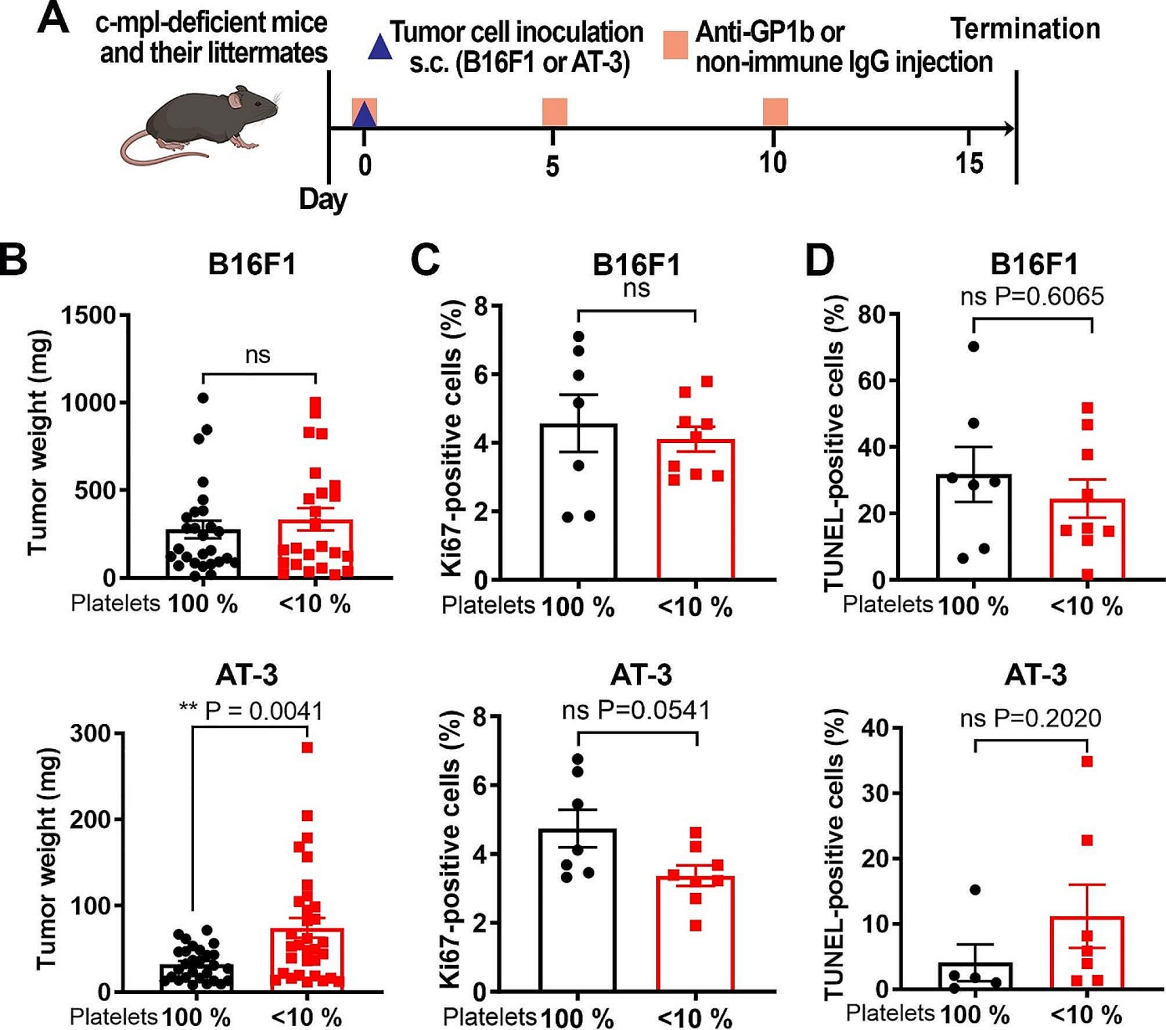
Impact of chronic severe thrombocytopenia on proliferation and apoptosis in B16F1 and AT-3 tumors. **(A)** Schematic representation of severe chronic thrombocytopenia induction. Starting from the day of B16F1 and AT-3 tumor cell implantation, c-mpl-deficient mice were injected every 5 days with a platelet-depleting polyclonal antibody at low dose (0.5 µg/g mouse). Littermate control mice were injected with a non-immune IgG. **(B)** Wet weight of B16F1 tumors (platelet 100%, *n* = 27; <10%, *n* = 24) and AT-3 tumors (platelet 100%, *n* = 28; <10%, *n* = 32). **(C)** Quantification of KI67-positive cells in B16F1 tumors and AT-3 tumors from control mice and mice with chronic severe thrombocytopenia. *n* = 7 (100%) and 9 (< 10%) different B16F1 tumors; and *n* = 7 (100%) and 8 (< 10%) different AT-3 tumors. **(D)** Quantification of TUNEL-positive cells in B16F1 tumors and AT-3 tumors from control mice and mice with chronic severe thrombocytopenia mice. *n* = 7 (100%) and 9 (< 10%) different B16F1 tumors; and *n* = 5 (100%) and 7 (< 10%) different AT-3 tumors. ns: non-significant

The continuous presence of platelets in the TME of both B16F1 and AT-3 tumors raised the question of their impact on these tumors’ development and microenvironment shaping. In order to address this question, we implanted B16F1 and AT-3 tumors in mice lacking Mpl (c-mpl^−/−^ mice), the receptor for thrombopoietin, a key regulator of platelet production. These mice are characterized by constitutive thrombocytopenia, with an 80 to 90% reduction in their megakaryocyte and platelet numbers [22, 33]. Yet, because in mice most platelet functions are maintained for platelet counts over 10% [34, 35], residual platelets in c-mpl^−/−^ mice were eliminated by repeated injections of a platelet depleting antibody used at low dose (Fig. 7A).

With regard to intratumor platelets, B16F1 and AT-3 tumors from thrombocytopenic mice were devoid of intra- and perivascular platelets. Strikingly, however, stromal clusters of extravascular platelets were still present in AT-3 tumors from thrombocytopenic mice (Fig. 6A-B), suggesting that these platelets are produced irrespective of the thrombopoietin/Mpl pathway and that their lineage might be distinct from that of circulating platelets.

**Fig. 8:**
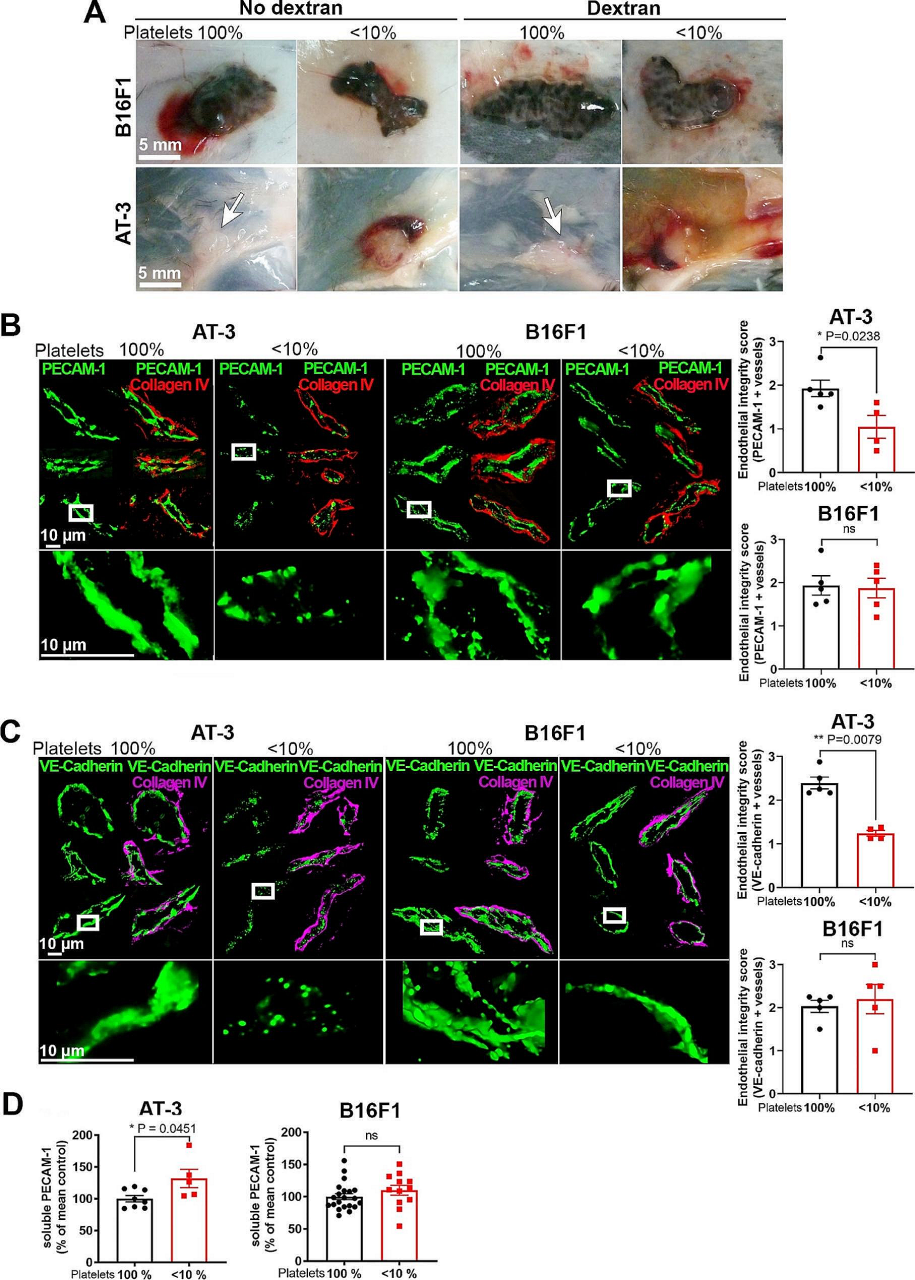
Impact of thrombocytopenia on vascular permeability and endothelial integrity in B16F1 and AT-3 tumors. **A.** Representative images of the macroscopic aspects of B16F1 and AT-3 tumors from control mice and mice with chronic severe thrombocytopenia, with or without intravenous injection of FITC-dextran, 2000 kDa prior to sacrifice. AT-3 tumors in control mice are highlighted by white arrows. **B-C.** Representative images and corresponding endothelial integrity scores of PECAM-1 (**B**) and VE-cadherin (**C**) staining for evaluation of endothelial integrity in AT-3 and B16F1 tumors from control mice and mice with chronic severe thrombocytopenia. Maximal intensity projections of optical sections are shown. **D.** Comparison of soluble PECAM-1 levels in plasma from control mice and mice with chronic severe thrombocytopenia with AT-3 (platelet 100%, *n* = 8; <10%, *n* = 5) or B16F1 (platelet 100%, *n* = 21; <10%, *n* = 12) tumors. ns: non-significant

After 15 days of tumor development, there were no differences in tumor weight, proliferation index, and apoptotic index between B16F1 tumors from control and thrombocytopenic mice (Fig. 7B-C). In contrast, AT-3 tumors grown in mice with severe chronic thrombocytopenia were significantly larger compared to those from mice with normal platelet counts (Fig. 7B). In agreement with previous studies showing that platelets prevent tumor bleeding [11, 18], intratumor hemoglobin content was significantly higher in thrombocytopenic mice compared to control mice, irrespective of the tumor type (Supplementary Fig. 9). Despite being larger, AT-3 tumors from thrombocytopenic mice showed a non-significant trend towards a decreased cell proliferation index (*p* = 0.0541), and no change in apoptotic cell index compared to AT-3 tumors from mice with normal platelet count (Fig. 7C-D). There was no difference in blood vessel density between control and thrombocytopenic mice, in either type of tumor model (Supplementary Fig. 10). The fact that AT-3 tumors grown in thrombocytopenic mice had an increased wet weight in spite of a similar vessel density and apoptotic index, and reduced proliferation index, compared to control tumors, suggested a possible increase in their tumor vessel permeability. At 1 h after intravenous injection of high molecular weight (2,000 kDa) FITC-dextran, massive, macroscopically visible peritumoral leakage of FITC-dextran was observed around AT-3 tumors from thrombocytopenic mice, but not from mice with normal platelet counts, and neither around B16F1 tumors from either control or thrombocytopenic mice (Fig. 8A). Immunostainings for PECAM-1 (Fig. 8B), VE-cadherin (Fig. 8C), and ZO-1 (Supplementary Fig. 11), all gave concordant results, revealing major alterations of the tumor vessel endothelium, specifically in AT-3 tumors from thrombocytopenic mice. These alterations included large discontinuities in the endothelial lining and signs of endothelial degeneration (Fig. 8B-C). Tumor vessel endothelial abnormalities in thrombocytopenic mice bearing AT-3 were associated with an increase in plasma PECAM-1 as compared to AT-3-bearing control mice (Fig. 8D). In contrast, the tumor vessel endothelial lining was homogeneous and continuous in all other mouse groups. Moreover, control and thrombocytopenic mice with B16F1 tumors had similar tumor endothelial integrity scores and plasma PECAM-1 levels (Fig. 8B-D). Importantly, neither the increase in tumor weight, nor the endothelial abnormalities and increased permeability of AT-3 tumors were observed in c-mpl^−/−^ mice that had not been treated with the platelet-depleting antibody for elimination of residual circulating platelets (Supplementary Fig. 12). In a similar manner, none of the tumor vessel features of AT-3 tumors from mice with chronic severe thrombocytopenia were observed in mice with a genetic deficiency in platelet GPVI (Supplementary Fig. 13), a receptor which has been shown previously to intervene in the prevention of bleeding in inflamed organs and tumors [31, 36–39].

### Platelets exert tumor-type specific immunomodulatory functions

**Fig. 9:**
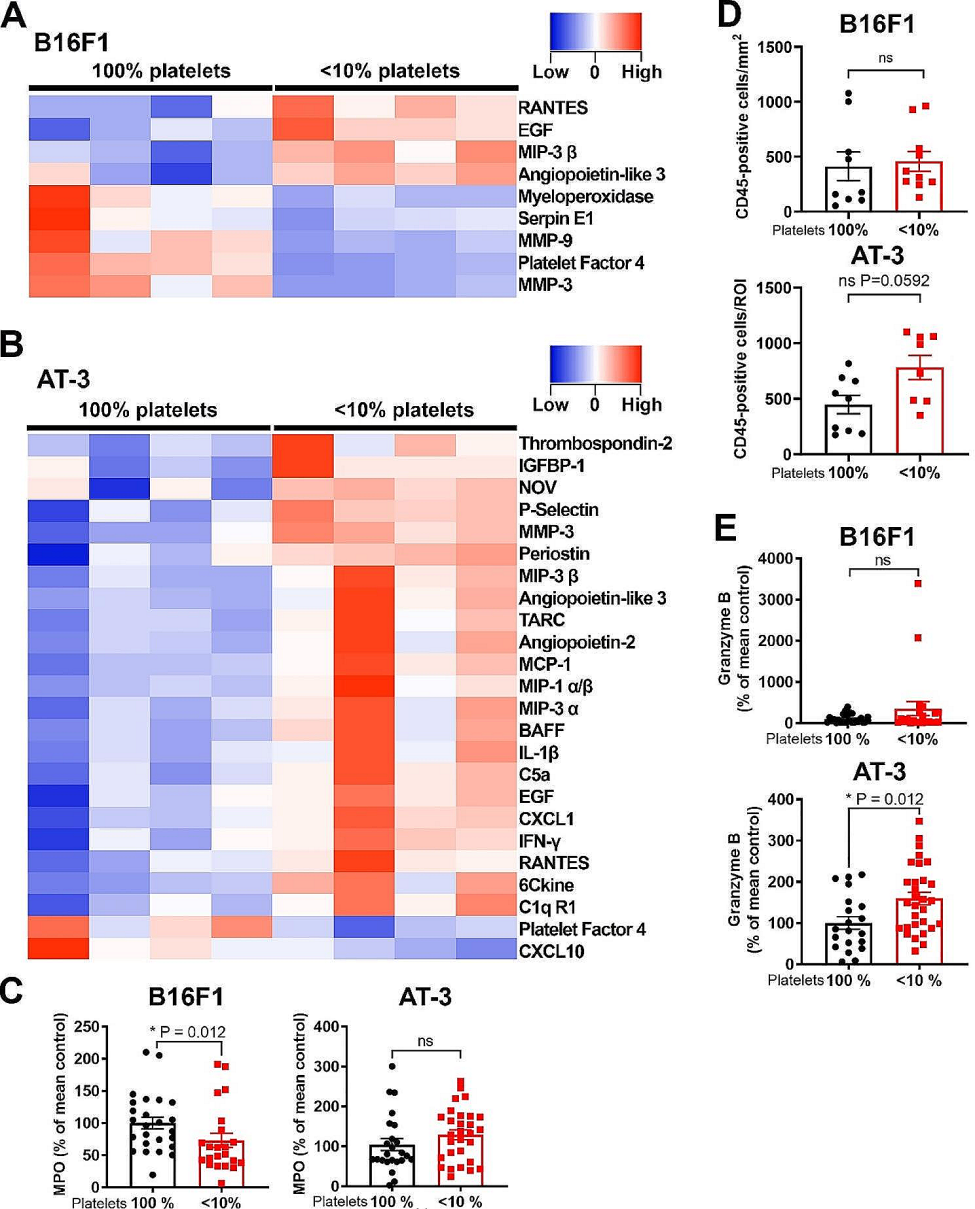
Impact of chronic severe thrombocytopenia on the inflammatory and immune profiles of B16F1 and AT-3 tumors. A-B. Comparative heatmap of tumor cytokines and angiogenic factors expressed in (**A**) B16F1 and (**B**) AT-3 tumor extracts from control mice and mice with chronic severe thrombocytopenia mice using Proteome Profiler arrays (*n* = 4 tumors per group, *p* < 0.05). **C.** Comparison of myeloperoxidase (MPO) levels used as a marker of neutrophil infiltration in B16F1 and AT-3 tumor extracts from control mice and mice with chronic severe thrombocytopenia. *n* = 25 (100%) and 22 (< 10%) B16F1 tumors; *n* = 24 (100%) and 29 (< 10%) AT-3 tumors. **D.** Quantification of CD45-positive cells in B16F1 and AT-3 tumors from control mice and mice with chronic severe thrombocytopenia. *n* = 9 (100%) and 10 (< 10%) different B16F1 tumors; and *n* = 9 (100%) and 8 (< 10%) different AT-3 tumors. **E.** Comparison of cytotoxic cell infiltration among control mice and mice with chronic severe thrombocytopenia in B16F1 and AT-3 tumors by measurement of their granzyme B content. *n* = 25 (100%) and 22 (< 10%) different B16F1 tumors; and *n* = 20 (100%) and 30 (< 10%) different AT-3 tumors. ns: non-significant

In order to determine whether platelets participate in the regulation of the inflammatory and immune environment of B16F1 and AT-3 tumors, we compared the cytokine proteome profile of tumors grown in control and thrombocytopenic mice. Thrombocytopenia modified the cytokine proteome profile of both tumor types, with a more pronounced impact on that of AT-3 tumors. Thrombocytopenia indeed caused significant changes in the intratumor levels of 9 cytokines or angiogenesis-related proteins in B16F1 tumors vs. 24 for AT-3 tumors (Fig. 9A and Supplementary Fig. 1). Thrombocytopenia-associated changes common to both tumor types included an increase in RANTES, EGF, and angiopoietin-like 3 levels, and a reduction in PF4 levels (Fig. 8A). Thrombocytopenia led to a significant reduction in intratumor PAI-1, MMP-3, MMP-9, and MPO, specifically in B16F1 tumors (Fig. 9A). The reduced intratumor MPO content of B16F1 tumors grown in thrombocytopenic mice was confirmed by ELISA on a larger sample set (Fig. 9C). In AT-3 tumors, thrombocytopenia caused a significant reduction in the levels of PF4 and CXCL-10, and an increase in those of several major mediators of immune cell recruitment and activation, including MCP-1, IFN-γ, RANTES, P-selectin, macrophage inflammatory protein-1 α and β (MIP-1α/β), MIP-3β, interleukin-1β (IL-1β) and CXC chemokine KC (CXCL1) (Fig. 9A).

Regarding tumor-infiltrating leukocytes, thrombocytopenia did not affect the overall content of intratumor CD45 + cells in B16F1 tumors whereas a non-significant trend towards an increased intratumor CD45 + cells was observed in AT-3 tumors (*p* = 0.0592). (Fig. 9D), thus suggesting a differential impact of platelets on leukocyte infiltration depending on the tumor type. In line with this result, thrombocytopenia also resulted in a different outcome between B16F1 and AT-3 tumors with respect to intratumor cytotoxic cell content, as estimated by measurement of granzyme B level. In fact, whereas thrombocytopenia did not affect intratumor granzyme B level in B16F1 tumors (Fig. 9E), it increased it in AT-3 tumors (Fig. 9E).

## Discussion

Here, we investigated the participation of platelets in the TME of two types of solid tumors displaying drastically different vascular and inflammatory microenvironments. Among the main TME-related differences between B16F1 and AT-3 tumors, AT-3 mammary tumors were more inflammatory than B16F1 melanoma tumors, as indicated by their increased content in immune cells and mediators of immune cell recruitment. It is worth noting that despite their increased immune cell content, which included an increased cytotoxic cell content, AT-3 tumors presented signs of decreased sensitivity to antitumor immunity compared to B16F1 melanoma [40]. In fact, whereas a negative correlation between tumor granzyme B content and tumor weight was found in B16F1 tumors, there was no association between granzyme B content and tumor weight in AT-3 tumors. The uncoupling of cytotoxic cell content and tumor growth in AT-3 tumors may be linked to their increased content in PD-L1. Indeed, PD-L1 was previously shown to suppress NK and CD8 T cell antitumor activity [41, 42]. Importantly, our result show that, as different as AT-3 and B16F1 tumors are, platelets are constitutive components of their respective TME. Irrespective of thrombosis and bleeding, platelets were systematically present in the intravascular and perivascular compartments of both AT-3 and B16F1 tumors.

Intravital microscopy revealed that platelets were in constant interactions with tumor vessels, mainly under the form of firmly adherent non-aggregated platelets continuously lining the tumor vasculature. While the mechanisms underlying direct interactions between tumor cells and platelets have been extensively studied in vitro and in models of hematogenous metastasis [43, 44], there is only little information on if and how platelets interact with solid tumors in vivo. Twenty years ago, Manegold et al. provided the first in vivo demonstration that platelets interact with the tumor microcirculation [45]. They reported slightly increased rolling but no firm adhesion of platelets in tumor microvessels of mice bearing Lewis lung carcinoma or methylcholanthrene-induced fibrosarcoma [45]. The discrepancy regarding the presence of firmly adherent platelets in tumor microvessels between our study and this earlier study can be explained by the technological advances made in in vivo visualization of platelets since then. The infusion of a fluorescent antibody to platelets allowed us to visualize all circulating platelets, whereas only a minor subset of ex vivo labeled platelets could be visualized in the study by Manegold et al.

Evidence of sustained, local interactions between platelets and tumor vessels provides a mechanistic insight on how platelets deliver their growth factors and many other bioactive molecules into tumors to help shape the TME and tumor cell phenotype. It has been shown previously that platelets contribute to the recruitment of pericytes and cancer-associated fibroblasts in tumors and favor the epithelial-to-mesenchymal transition through the release of soluble factors like transforming growth factor beta (TGFβ) and platelet-derived growth factor B (PDGFB) from their secretion granules [2, 17, 46, 47]. While these effects of platelets and platelet secretion products have suggested physical interactions between platelets and tumors, the existence of such interactions in vivo remained to be demonstrated.

Firm adhesion of non-aggregated platelets to tumor vessels is also consistent with the prevention of bleeding in tumors by platelets. In agreement with previous studies [11, 18, 19], we show that thrombocytopenia increased bleeding in B16F1 and AT-3 tumors. Innate immune cells like neutrophils and macrophages have been shown to cause vessel injury and bleeding in tumors [11, 19]. Our results indicate that, as was shown in other inflammatory settings [48], non-aggregated platelets contribute to the sealing of immune cell-induced vessel injury in tumors. Beyond prevention of tumor bleeding, we show here that platelets further support tumor endothelial integrity in AT-3 tumors, an effect that was not observed in B16F1 tumors. Severe thrombocytopenia resulted in endothelial degeneration associated with massive vascular leakage, leading to significantly larger AT-3 tumors. The difference in thrombocytopenia-associated tumor endothelial phenotype between AT-3 and B16F1 tumors indicates that the absence of platelets per se is not sufficient to cause tumor endothelial degeneration, and stresses the involvement of additional, tumor type-related, local hits. It also indicates that the long-suspected endothelial-nurturing effect of platelets highlighted by early and more recent studies [49, 50], may become particularly relevant in certain tumor microenvironments.

Notably, the endothelial phenotype of AT-3 tumors from mice with chronic and severe thrombocytopenia was not observed in c-mpl^−/−^ mice, which have between 10 and 20% of residual circulating platelets. This indicates that those endothelial alterations are not due to c-mpl deficiency-related changes in bone marrow hematopoietic cells and that, like for prevention of inflammation-induced bleeding [48], a very limited number of platelets is sufficient to maintain endothelial integrity in AT-3 tumors. Likewise, we did not observe any bleeding or permeability defects in AT-3 or B16F1 tumors from GPVI^−/−^ mice. GPVI is the main platelet receptor for collagen and a mediator of fibrin-induced procoagulant activity of platelets [51–53]. It has been implicated in various platelet functions pertaining to the maintenance of vascular integrity, spanning from promotion of endothelial barrier function [49] to repair of neutrophil-induced injury [31, 36, 38, 39], including in solid tumors [11]. Whereas our results do not rule out a role for GPVI in the maintenance of tumor vascular integrity, they indicate that, in some tumors and TME, GPVI deficiency can be compensated. This might not be surprising as functional redundancy between platelet receptors and activation pathways has been shown to be highly effective for maintenance of vascular integrity in inflamed tissues [37, 48].

In contrast to tumor vessel function, tumor vessel density in B16F1 and AT-3 tumors remained unchanged by thrombocytopenia. Studies on the impact of platelets on tumor angiogenesis have yielded inconsistent results [8, 54, 55]. Our results indicate that, from a quantitative perspective, there are tumors in which angiogenesis can occur normally in the absence of platelets, and therefore suggest that the participation of platelets in tumor angiogenesis stimulation may depend on the tumor type.

Intriguingly, in addition to intravascular and perivascular platelets, we found an abundance of extravascular platelet clusters in the stroma of AT-3 tumors. The fact that extravascular platelet clusters were still present in AT-3 tumors from mice with severe thrombocytopenia resulting from c-mpl deficiency combined with antibody-mediated immunodepletion of circulating platelets raises questions about their origin. Whether these platelets result from extramedullar thrombopoietin-independent megakariopoiesis programs remains highly hypothetical, but it is worth noting that there is accumulating evidence of non-classical pathways for platelet production, particularly in inflammatory settings [56–59]. Moreover, several studies have shown that megakaryocytes and platelets can be produced from mesenchymal stem cells derived from adipose tissue of the mammary fat pads or subcutaneous layer [60–62]. Nonetheless, one cannot exclude that these extravascular platelet clusters were formed by the residual circulating platelets in mice with chronic severe thrombocytopenia.

Understanding how these mammary tumor-associated extravascular platelet clusters form could help determine their functions in future studies.

Platelets contain and secrete a plethora of immunomodulatory factors [63]. Nevertheless, data on the impact of platelets on tumor inflammation and antitumor immune response remain scarce. We show that platelets play a significant role in the regulation of the inflammatory profile and immune cell content of both B16F1 and AT-3 tumors, yet with very different outcomes according to the tumor type. Thrombocytopenia had a limited impact on the inflammatory profile of B16F1 tumors. It led mainly to a reduction in intratumor MPO content, indicative of reduced neutrophil infiltration, a result consistent with the ability of platelets to support neutrophil infiltration in a variety of inflamed tissues [63]. Thrombocytopenia was associated with important changes in the inflammatory profile of AT-3 tumors and led to a marked increase in their cytotoxic cell content. This result is consistent with those of a previous study showing that thrombocytopenia resulted in increased cytotoxic cell infiltration in experimental colon and bladder cancers [64], and suggests that, in certain tumors, platelets contribute to dampen spontaneous anticancer immunity. Interestingly, over the last years, several studies have shown that platelets from cancer patients can express PD-L1 [21, 65, 66].

## Conclusions

In summary, our findings indicate that platelets are integral components of the TME of two highly different solid tumor models, on which they exert differential effects. The different intratumor localizations and effects of platelets according to the tumor model suggest that the interest of antiplatelet therapy for chemoprevention and cancer treatment may vary greatly between solid tumors. Immunohistological testing for platelets during pathological examination in relation to clinical characteristics might provide a tool to help identify which cancers might benefit from antiplatelet strategies.

### Electronic supplementary material

Below is the link to the electronic supplementary material.

Supplementary Material 1

Supplementary Material 2

Supplementary Material 3

Supplementary Material 4

Supplementary Material 5

Supplementary Material 6

Supplementary Material 7

Supplementary Material 8

Supplementary Material 9

Supplementary Material 10

Supplementary Material 11

## Data Availability

Data and protocols will be made available to other investigators upon request to the corresponding author in consultation with all co-authors. **Declarations**. All procedures were approved by the local animal ethics committee registered with the French Ministry of Research (APAFIS project authorization#31821-2021052715257618). Not applicable. The authors declare that they have no competing interests.

## References

[1] Gasic GJ, Gasic TB, Stewart CC (1968). Antimetastatic effects associated with platelet reduction. Proc Natl Acad Sci U S A.

[2] Labelle M, Begum S, Hynes RO (2011). Direct signaling between platelets and Cancer cells induces an epithelial-mesenchymal-like Transition and promotes metastasis. Cancer Cell.

[3] Nieswandt B, Hafner M, Echtenacher B, Männel DN (1999). Lysis of tumor cells by natural killer cells in mice is impeded by platelets. Cancer Res.

[4] Kim YJ, Borsig L, Varki NM, Varki A (1998). P-selectin deficiency attenuates tumor growth and metastasis. Proc Natl Acad Sci U S A.

[5] Jain S, Zuka M, Liu J, Russell S, Dent J, Guerrero JA (2007). Platelet glycoprotein Ibα supports experimental lung metastasis. Proc Natl Acad Sci U S A.

[6] Jain S, Russell S, Ware J (2009). Platelet glycoprotein VI facilitates experimental lung metastasis in syngenic mouse models. J Thromb Haemost.

[7] Mezouar S, Darbousset R, Dignat-George F, Panicot-Dubois L, Dubois C (2015). Inhibition of platelet activation prevents the P-selectin and integrin-dependent accumulation of cancer cell microparticles and reduces tumor growth and metastasis in vivo. Int J Cancer.

[8] Stone RL, Nick AM, McNeish IA, Balkwill F, Han HD, Bottsford-Miller J (2012). Paraneoplastic thrombocytosis in Ovarian Cancer. N Engl J Med.

[9] Bottsford-Miller J, Choi HJ, Dalton HJ, Stone RL, Cho MS, Haemmerle M (2015). Differential platelet levels affect response to taxane-based therapy in ovarian cancer. Clin Cancer Res.

[10] Plantureux L, Mege D, Crescence L, Carminita E, Robert S, Cointe S (2020). The interaction of platelets with colorectal cancer cells inhibits tumor growth but promotes metastasis. Cancer Res.

[11] Volz J, Mammadova-Bach E, Gil-Pulido J, Nandigama R, Remer K, Sorokin L (2019). Inhibition of platelet GPVI induces intratumor hemorrhage and increases efficacy of chemotherapy in mice. Blood.

[12] Cho MS, Bottsford-Miller J, Vasquez HG, Stone R, Zand B, Kroll MH (2012). Platelets increase the proliferation of ovarian cancer cells. Blood.

[13] Michael Jv, Wurtzel JGT, Mao GF, Rao AK, Kolpakov MA, Sabri A (2017). Platelet microparticles infiltrating solid tumors transfer miRNAs that suppress tumor growth. Blood.

[14] Egan K, Crowley D, Smyth P, O’Toole S, Spillane C, Martin C et al. Platelet adhesion and Degranulation Induce Pro-survival and Pro-angiogenic Signalling in Ovarian Cancer cells. PLoS ONE. 2011;6. 10.1371/journal.pone.0026125 PMC3192146 22022533

[15] Ibele GM, Kay NE, Johnson GJ, Jacob HS (1985). Human platelets exert cytotoxic effects on tumor cells. Blood.

[16] Kisucka J, Butterfield CE, Duda DG, Eichenberger SC, Saffaripour S, Ware J (2006). Platelets and platelet adhesion support angiogenesis while preventing excessive hemorrhage. Proc Natl Acad Sci U S A.

[17] Zhang Y, Cedervall J, Hamidi A, Herre M, Viitaniemi K, D’Amico G (2020). Platelet-specific PDGFB ablation impairs Tumor Vessel Integrity and promotes metastasis. Cancer Res.

[18] Ho-Tin-Noé B, Goerge T, Cifuni SM, Duerschmied D, Wagner DD (2008). Platelet granule secretion continuously prevents intratumor hemorrhage. Cancer Res.

[19] Ho-Tin-Noé B, Carbo C, Demers M, Cifuni SM, Goerge T, Wagner DD (2009). Innate Immune cells induce hemorrhage in tumors during Thrombocytopenia. Am J Pathol.

[20] Pavlović N, Kopsida M, Gerwins P, Heindryckx F (2021). Activated platelets contribute to the progression of hepatocellular carcinoma by altering the tumor environment. Life Sci.

[21] Hinterleitner C, Strähle J, Malenke E, Hinterleitner M, Henning M, Seehawer M et al. Platelet PD-L1 reflects collective intratumoral PD-L1 expression and predicts immunotherapy response in non-small cell lung cancer. Nat Commun. 2021;12. 10.1038/s41467-021-27303-7 PMC8636618 34853305

[22] Gurney AL, Carver-Moore K, De Sauvage FJ, Moore MW (1994). Thrombocytopenia in c-mpl-deficient mice. Science.

[23] Lockyer S, Okuyama K, Begum S, Le S, Sun B, Watanabe T (2006). GPVI-deficient mice lack collagen responses and are protected against experimentally induced pulmonary thromboembolism. Thromb Res.

[24] Fidler IJ (1975). Biological behavior of malignant melanoma cells correlated to their survival in vivo. Cancer Res.

[25] Stewart TJ, Abrams SI (2007). Altered Immune function during long-term host-tumor interactions can be modulated to Retard Autochthonous neoplastic growth. J Immunol.

[26] Guy CT, Cardiff RD, Muller WJ (1992). Induction of mammary tumors by expression of polyomavirus middle T oncogene: a transgenic mouse model for metastatic disease. Mol Cell Biol.

[27] Lin EY, Jones JG, Li P, Zhu L, Whitney KD, Muller WJ (2003). Progression to Malignancy in the Polyoma Middle T Oncoprotein mouse breast Cancer Model provides a Reliable Model for Human diseases. Am J Pathol.

[28] Pfefferle AD, Herschkowitz JI, Usary J, Harrell JC, Spike BT, Adams JR (2013). Transcriptomic classification of genetically engineered mouse models of breast cancer identifies human subtype counterparts. Genome Biol.

[29] Attalla S, Taifour T, Bui T, Muller W (2021). Insights from transgenic mouse models of PyMT-induced breast cancer: recapitulating human breast cancer progression in vivo. Oncogene.

[30] Leunig M, Yuan F, Menger MD, Boucher Y, Goetz AE, Messmer K (1992). Angiogenesis, Microvascular Architecture, Microhemodynamics, and interstitial fluid pressure during early growth of human adenocarcinoma LS174T in SCID mice. Cancer Res.

[31] Lockyer S, Okuyama K, Begum S, Le S, Sun B, Watanabe T (2015). Single platelets seal neutrophil-induced vascular breaches via GPVI during immune-complex-mediated inflammation in mice. Blood.

[32] Wendel M, Galani IE, Suri-Payer E, Cerwenka A (2008). Natural killer cell accumulation in tumors is dependent on IFN-gamma and CXCR3 ligands. Cancer Res.

[33] Alexander WS, Roberts AW, Nicola NA, Li R, Metcalf D (1996). Deficiencies in Progenitor cells of multiple hematopoietic lineages and defective megakaryocytopoiesis in mice lacking the Thrombopoietin receptor c-Mpl. Blood.

[34] Morowski M, Vögtle T, Kraft P, Kleinschnitz C, Stoll G, Nieswandt B (2013). Only severe thrombocytopenia results in bleeding and defective thrombus formation in mice. Blood.

[35] Goerge T, Ho-Tin-Noe B, Carbo C, Benarafa C, Remold-O’Donnell E, Zhao BQ (2008). Inflammation induces hemorrhage in thrombocytopenia. Blood.

[36] Boulaftali Y, Hess PR, Getz TM, Cholka A, Stolla M, Mackman N (2013). Platelet ITAM signaling is critical for vascular integrity in inflammation. J Clin Invest.

[37] Rayes J, Jadoui S, Lax S, Gros A, Wichaiyo S, Ollivier V (2018). The contribution of platelet glycoprotein receptors to inflammatory bleeding prevention is stimulus and organ dependent. Haematologica.

[38] Kaiser R, Escaig R, Kranich J, Hoffknecht ML, Anjum A, Polewka V (2022). Procoagulant platelet sentinels prevent inflammatory bleeding through GPIIBIIIA and GPVI. Blood.

[39] Currie SM, Stegmeyer RI, Mildner K, Breitsprecher L, Zeuschner D, Psathaki OE (2022). Confocal Real-Time Analysis of Cutaneous Platelet Recruitment during Immune complex–mediated inflammation. J Invest Dermatology.

[40] Cullen SP, Brunet M, Martin SJ (2010). Granzymes in cancer and immunity. Cell Death Differ.

[41] Hsu J, Hodgins JJ, Marathe M, Nicolai CJ, Bourgeois-Daigneault MC, Trevino TN (2018). Contribution of NK cells to immunotherapy mediated by PD-1/PD-L1 blockade. J Clin Invest.

[42] Juneja VR, McGuire KA, Manguso RT, LaFleur MW, Collins N, Nicholas Haining W (2017). PD-L1 on tumor cells is sufficient for immune evasion in immunogenic tumors and inhibits CD8 T cell cytotoxicity. J Exp Med.

[43] Schlesinger M. Role of platelets and platelet receptors in cancer metastasis. J Hematol Oncol. 2018;11. 10.1186/s13045-018-0669-2 PMC6180572 30305116

[44] Le Chapelain O, Ho-Tin-noé B (2022). Intratumoral platelets: harmful or incidental bystanders of the Tumor Microenvironment?. Cancers (Basel).

[45] Manegold PC, Hutter J, Pahernik SA, Messmer K, Dellian M (2003). Platelet-endothelial interaction in tumor angiogenesis and microcirculation. Blood.

[46] Guo Y, Cui W, Pei Y, Xu D (2019). Platelets promote invasion and induce epithelial to mesenchymal transition in ovarian cancer cells by TGF-β signaling pathway. Gynecol Oncol.

[47] Zhang Y, Manouchehri Doulabi E, Herre M, Cedervall J, Qiao Q, Miao Z (2022). Platelet-derived PDGFB promotes recruitment of Cancer-Associated fibroblasts, deposition of extracellular matrix and Tgfβ signaling in the Tumor Microenvironment. Cancers (Basel).

[48] Ho-Tin-Noé B, Boulaftali Y, Camerer E (2018). Platelets and vascular integrity: how platelets prevent bleeding in inflammation. Blood.

[49] Gupta S, Konradt C, Corken A, Ware J, Nieswandt B, Di Paola J (2020). Hemostasis vs. homeostasis: platelets are essential for preserving vascular barrier function in the absence of injury or inflammation. Proc Natl Acad Sci U S A.

[50] Ho-Tin-Noé B, Le Chapelain O, Camerer E (2021). Platelets maintain vascular barrier function in the absence of injury or inflammation. J Thromb Haemost.

[51] Alshehri OM, Hughes CE, Montague S, Watson SK, Frampton J, Bender M (2015). Fibrin activates GPVI in human and mouse platelets. Blood.

[52] Mammadova-Bach E, Ollivier V, Loyau S, Schaff M, Dumont B, Favier R (2015). Platelet glycoprotein VI binds to polymerized fibrin and promotes thrombin generation. Blood.

[53] Gauer JS, Duval C, Xu RG, Macrae FL, McPherson HR, Tiede C (2023). Fibrin-glycoprotein VI interaction increases platelet procoagulant activity and impacts clot structure. J Thromb Haemost.

[54] Brockmann MA, Bender B, Plaxina E, Nolte I, Erber R, Lamszus K (2011). Differential effects of tumor-platelet interaction in vitro and in vivo in glioblastoma. J Neurooncol.

[55] Li R, Ren M, Chen N, Luo M, Deng X, Xia J et al. Presence of intratumoral platelets is associated with tumor vessel structure and metastasis. BMC Cancer. 2014;14. 10.1186/1471-2407-14-167 PMC4016490 24606812

[56] Haas S, Hansson J, Klimmeck D, Loeffler D, Velten L, Uckelmann H (2015). Inflammation-Induced Emergency Megakaryopoiesis Driven by hematopoietic stem cell-like megakaryocyte progenitors. Cell Stem Cell.

[57] Nishimura S, Nagasaki M, Kunishima S, Sawaguchi A, Sakata A, Sakaguchi H (2015). IL-1α induces thrombopoiesis through megakaryocyte rupture in response to acute platelet needs. J Cell Biol.

[58] Couldwell G, Machlus KR (2019). Modulation of megakaryopoiesis and platelet production during inflammation. Thromb Res.

[59] Morodomi Y, Kanaji S, Sullivan BM, Zarpellon A, Orje JN, Won E (2022). Inflammatory platelet production stimulated by tyrosyl-tRNA synthetase mimicking viral infection. Proc Natl Acad Sci U S A.

[60] Matsubara Y, Murata M, Ikeda Y (2012). Culture of megakaryocytes and platelets from subcutaneous adipose tissue and a preadipocyte cell line. Methods Mol Biol.

[61] Zhang J, Zhou S, Zhou Y, Feng F, Wang Q, Zhu X (2016). Adipose-derived mesenchymal stem cells (ADSCs) with the potential to ameliorate platelet recovery, enhance megakaryopoiesis, and inhibit apoptosis of bone marrow cells in a mouse model of Radiation-Induced Thrombocytopenia. Cell Transpl.

[62] Tozawa K, Ono-Uruga Y, Yazawa M, Mori T, Murata M, Okamoto S (2019). Megakaryocytes and platelets from a novel human adipose tissue-derived mesenchymal stem cell line. Blood.

[63] Gros A, Ollivier V, Ho-Tin-Noé B (2015). Platelets in inflammation: regulation of leukocyte activities and vascular repair. Front Immunol.

[64] Riesenberg BP, Ansa-Addo EA, Gutierrez J, Timmers CD, Liu B, Li Z (2019). Cutting Edge: Targeting thrombocytes to rewire anticancer immunity in the Tumor Microenvironment and Potentiate Efficacy of PD-1 blockade. J Immunol.

[65] Zaslavsky AB, Adams MP, Cao X, Maj T, Choi JE, Stangl-Kremser J (2020). Platelet PD-L1 suppresses anti-cancer immune cell activity in PD-L1 negative tumors. Sci Rep 2020.

[66] Darga EP, Dolce EM, Fang F, Kidwell KM, Gersch CL, Kregel S et al. PD-L1 expression on circulating tumor cells and platelets in patients with metastatic breast cancer. PLoS ONE. 2021;16. 10.1371/journal.pone.0260124 PMC8592410 34780566

