# Supplementary material for: The localization, origin, and impact of platelets in the tumor microenvironment are tumor type-dependent
Source: J Exp Clin Cancer Res. 2024 Mar 16;43:84. doi: 10.1186/s13046-024-03001-2 (PMC10944607; doi:10.1186/s13046-024-03001-2)
Supplement: Supplementary file 7 — Supplementary Material 7 [file 13046_2024_3001_MOESM7_ESM.docx]

**Supplemental Data**

**Detailed Material and Methods**

**Blood cell count evolution**

Blood was collected in EDTA from the retro-orbital venous plexus under 3% isoflurane anesthesia and analyzed using an automated counter (ABC Vet, Scil).

**Preparation of platelet poor plasma and tumor extracts**

Platelet-poor plasma (PPP) was prepared by centrifuging heparin-anticoagulated blood twice (1000 g for 5 min, 2000 g for 5 min with PGI2 (2 μg/mL) and apyrase (0,15 U/mL). Tumors were excised, weighed, homogenized in PBS supplemented with protease inhibitor cocktail (P8340, Sigma-Aldrich) at a volume-to-weight ratio of 5 μL/mg tumor, subjected to 3 freeze/thaw cycles and sonication, centrifuged at 5000 g for 5 minutes, and the resulting tumor extracts were collected and stored at -80°C.

**Immunohistology of tumors**

# Tumors were harvested after intracardiac perfusion with zinc fixative [1], fixed in the same buffer for 48 hours, embedded in paraffin, and sectioned at 5 μm. Tumors sections were stained with H&E. Immunostainings were performed as described previously [1], using primary antibodies to CD45 (clone 30-F11; Biolegend), collagen IV (PA1-26148, ThermoFischer Scientific), PECAM-1 (clone MEC 13.3; BD Pharmingen), VE-Cadherin (AF1002, R&D System), ZO-1 (61-7300; Thermoscientific), PF4 (500-P05, Peprotech), anti-GPIX (M051-0, Emfret), VWF (A0082, DAKO), CD42b (R300, Emfret), and Ki67 (ab6326, Abcam). Apoptotic cells were detected by terminal deoxynucleotidyl transferase mediated dUTP nick end labeling (TUNEL, Roche Applied Science). Tissue sections incubated with irrelevant IgG or no primary antibodies were used as negative controls. Tissue sections were counterstained with Hoechst 33342 (Life Technologies) and mounted in aqueous Fluorescent Mounting Medium (Ibidi). Images were acquired using a fluorescence microscope (Leica DMi8) and LasX software and analyzed with Fiji. For H&E staining, slides were mounted with DPX Mountant (Fluka BioChemika, Buchs, Switzerland) and images were acquired using the NanoZoomer Digital slide scanner (Hamamatsu Photonics).

**Quantitative analysis of immunohistological stainings**

Leukocyte infiltration and blood vessel density were estimated by counting CD45-positive cells and collagen IV-positive vessels in 5 random fields (10x objective) per tumor, respectively. The proliferative and apoptotic indexes were determined as the percentages of KI67- and TUNEL-positive nuclei relative to all DAPI-stained nuclei calculated from 10 random fields per tumor (acquired with a 40x objective for KI67, 10X objective for TUNEL). Endothelial integrity was evaluated by scoring the continuity of PECAM-1, VE-cadherin, ZO-1 staining on a scale from 0 (highly discontinuous or absence of staining) to 3 (continuous staining). Scoring was performed by 3 independent observers blind to the treatment and mouse genotype on 3 images taken from random fields (63x objective) from 4-5 tumors per group.

**Intravital microscopy**

B16F1 or AT-3 cells (1x10^6^) were injected subcutaneously in the dorsal skin and allowed to grow for up to 10 days before surgical implantation of a dorsal skinfold chamber [2] (APJ Trading Co) the day of intravital imaging. Mice were injected intravenously with Alexa Fluor-conjugated antibodies (0.4 μg/g mouse) to GPIX (Emfret Analytics), fibrin, Gr-1, and PECAM-1, and/or FITC-dextran (2000 kDa, 4 μg/g mouse). Observations were made under anesthesia with 1.5% isoflurane, and images were acquired using a fluorescence macroscope (MacroFluo, Leica) equipped with a thermostated heating plate, and a sCMOS camera (Orca-Flash-4.0, Hamamatsu Photonics).

**Determination of soluble PECAM-1 levels**

A polyclonal antibody to mouse PECAM-1 (R&D, #AF3628) was immobilized onto COOH-magnetic beads (MC10035-01, BioRad). PECAM-1 capture beads were then incubated with plasma samples for one hour at room temperature. After washing, beads-associated PECAM-1 was revealed using a biotinylated rat monoclonal antibody to mouse PECAM-1 (BD, clone MEC13.3), followed by incubation with streptavidin-phycoerythrin complex. Median fluorescent intensity of the beads was analyzed using a BioPlex 200® System (BioRad).

**Supplemental References**

1. Ho-Tin-Noé B, Carbo C, Demers M, Cifuni SM, Goerge T, Wagner DD. Innate Immune Cells Induce Hemorrhage in Tumors during Thrombocytopenia. Am J Pathol. 2009;175:1699–708.

2. Leunig M, Yuan F, Menger MD, Boucher Y, Goetz AE, Messmer K, et al. Angiogenesis, Microvascular Architecture, Microhemodynamics, and Interstitial Fluid Pressure during Early Growth of Human Adenocarcinoma LS174T in SCID Mice. Cancer Res. 1992;52:6553–60.

**Supplementary Figures**

**
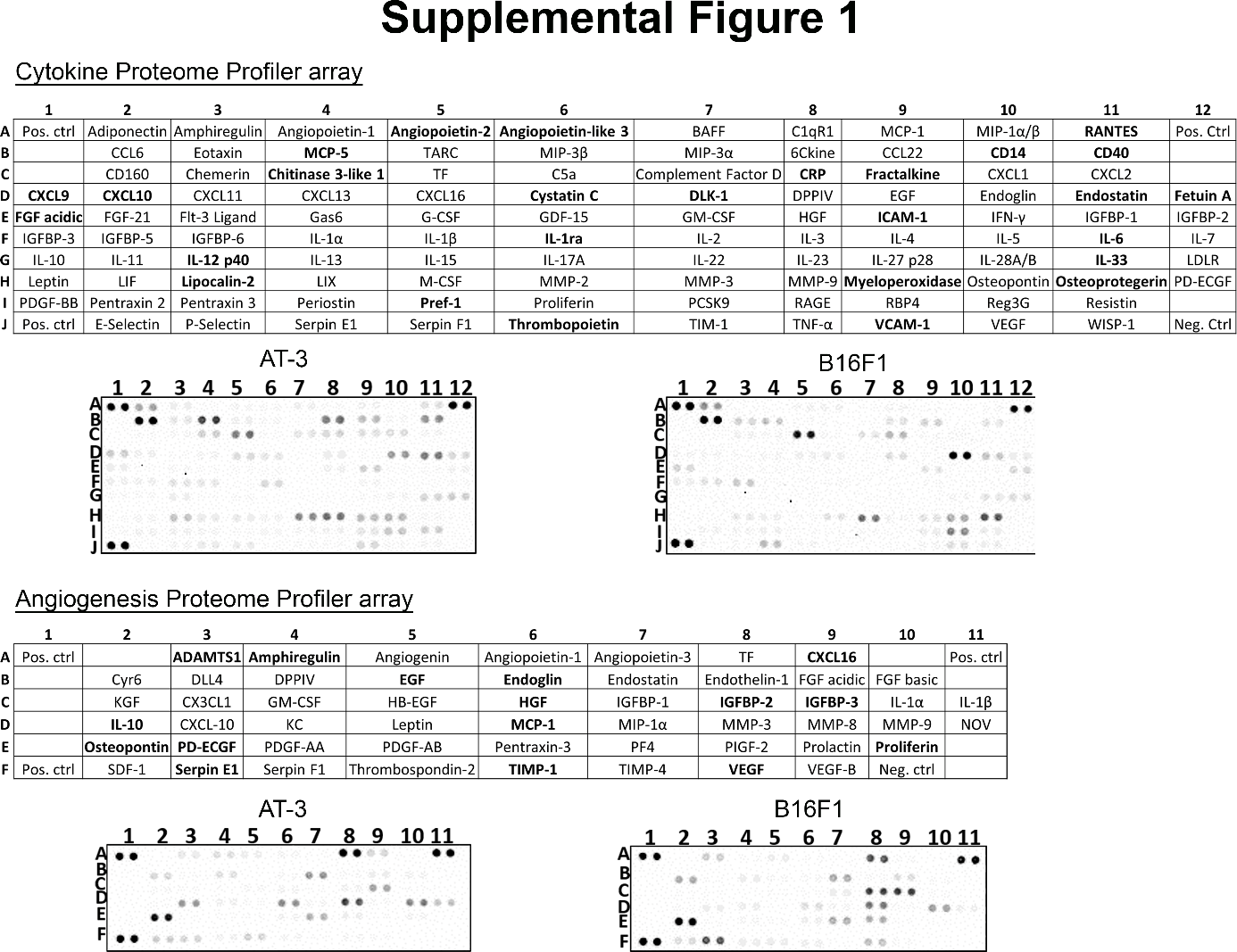
**

**Supplementary Figure 1. Cytokine and angiogenic factor expression using Proteome Profiler arrays (R&S Systems).** List and location of proteins analyzed on cytokine and angiogenic array membranes, and representative pictures of membranes probed for AT-3 and B16F1 tumor extracts. Proteins showing statistically significant differences are highlighted in bold text.

**
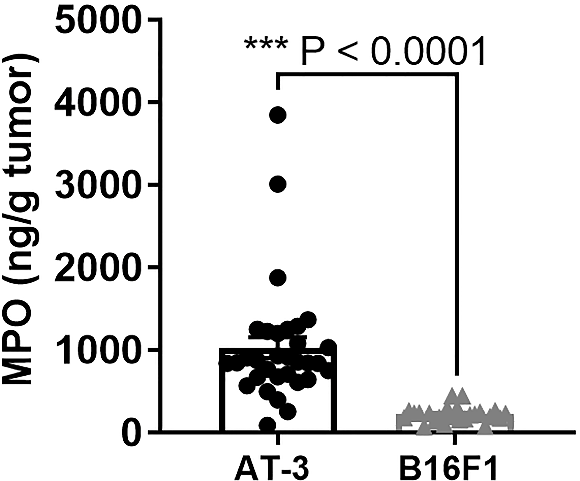
**

**Supplementary Figure 2. Comparison of intratumor myeloperoxidase content between AT-3 and B16F1 tumors.** Myeloperoxydase (MPO) levels in AT-3 (n=32) and B16F1 tumor extracts (n=25), as evaluated by ELISA.

**
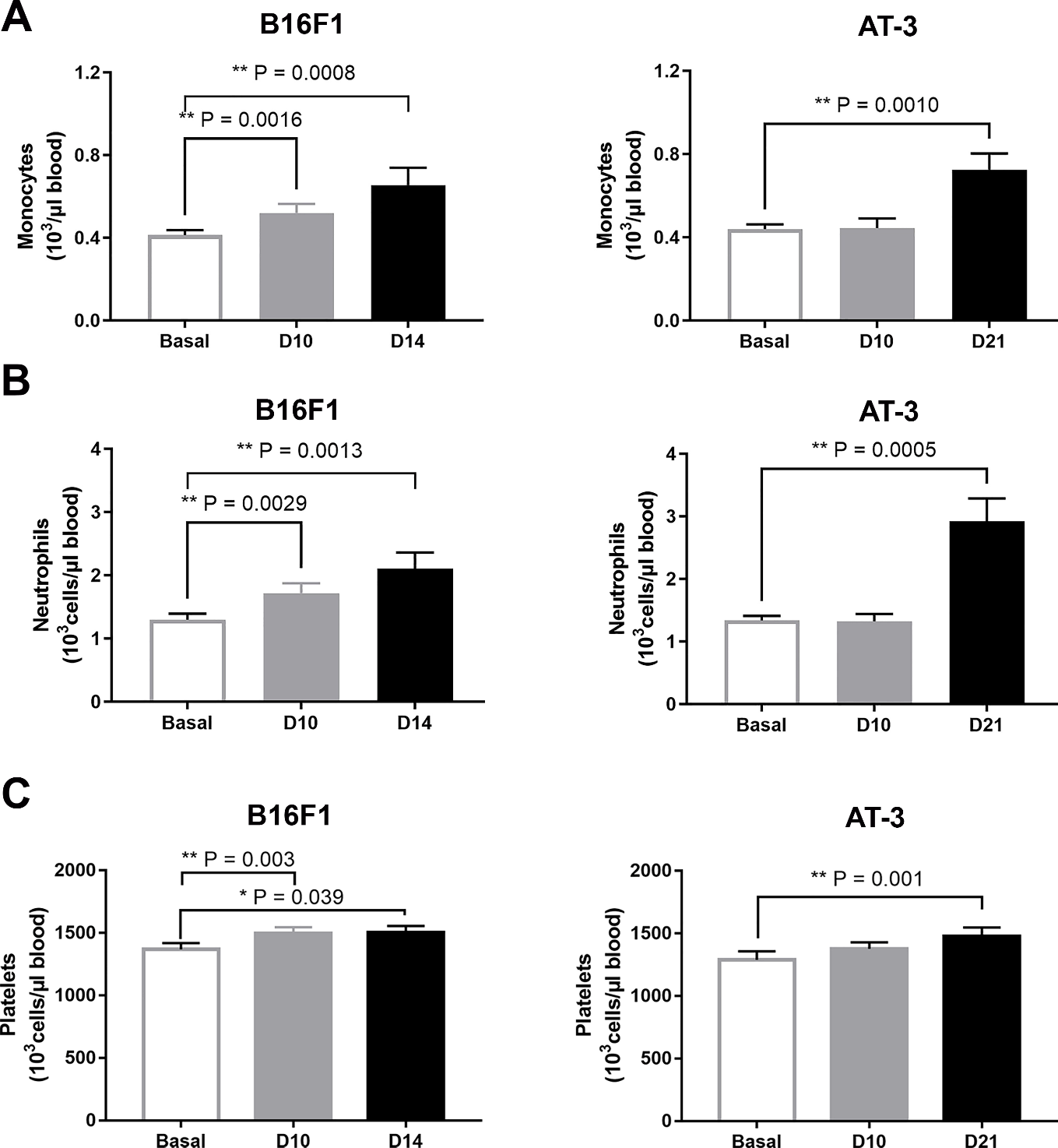
**

**Supplementary Figure 3. Evolution of monocyte, neutrophil and platelet counts in B16F1 and AT-3 tumor-bearing mice. A-C.** Evolution of blood monocyte (**A**), neutrophil (**B**) and platelet (**C**) counts following implantation of B16F1 and AT-3 cells. n=29 mice with B16F1 tumors; n =12 mice with AT-3 tumors.


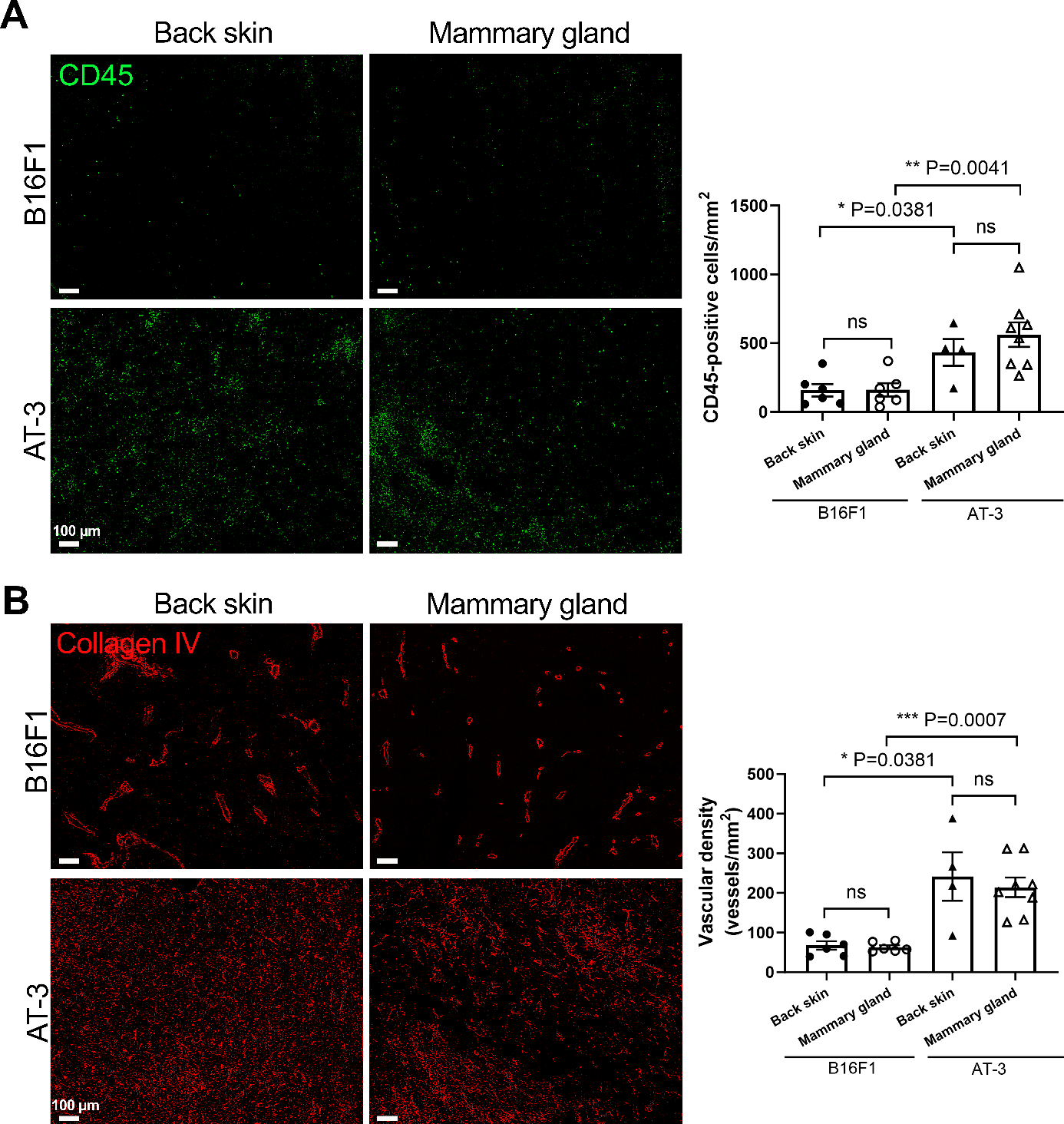


**Supplementary Figure 4. Differences in vessel density and leukocyte infiltration between AT-3 and B16F1 tumors are maintained irrespective of the implantation site and experiment length. A.** Representative images and corresponding quantification of CD45+ cell infiltration in AT-3 and B16F1 tumors grown either in the back skin (left panels) or mammary gland (right panels) of female mice (n=8 mammary and 4 back skin AT-3 tumors; n= 6 mammary and 6 back skin B16F1 tumors). Each dot represents the mean value calculated from 5 random fields taken with a 10x objective for a given tumor. **B.** Representative images and corresponding quantifications of collagen IV+ vessels in AT-3 and B16F1 tumors grown either in the back skin (left panels) or mammary gland (right panels) of female mice (n=8 mammary and 4 back skin AT-3 tumors; n= 6 mammary and 6 back skin B16F1 tumors). Each dot represents the mean value calculated from 5 random fields taken with a 10x objective for a given tumor. ns: non-significant.


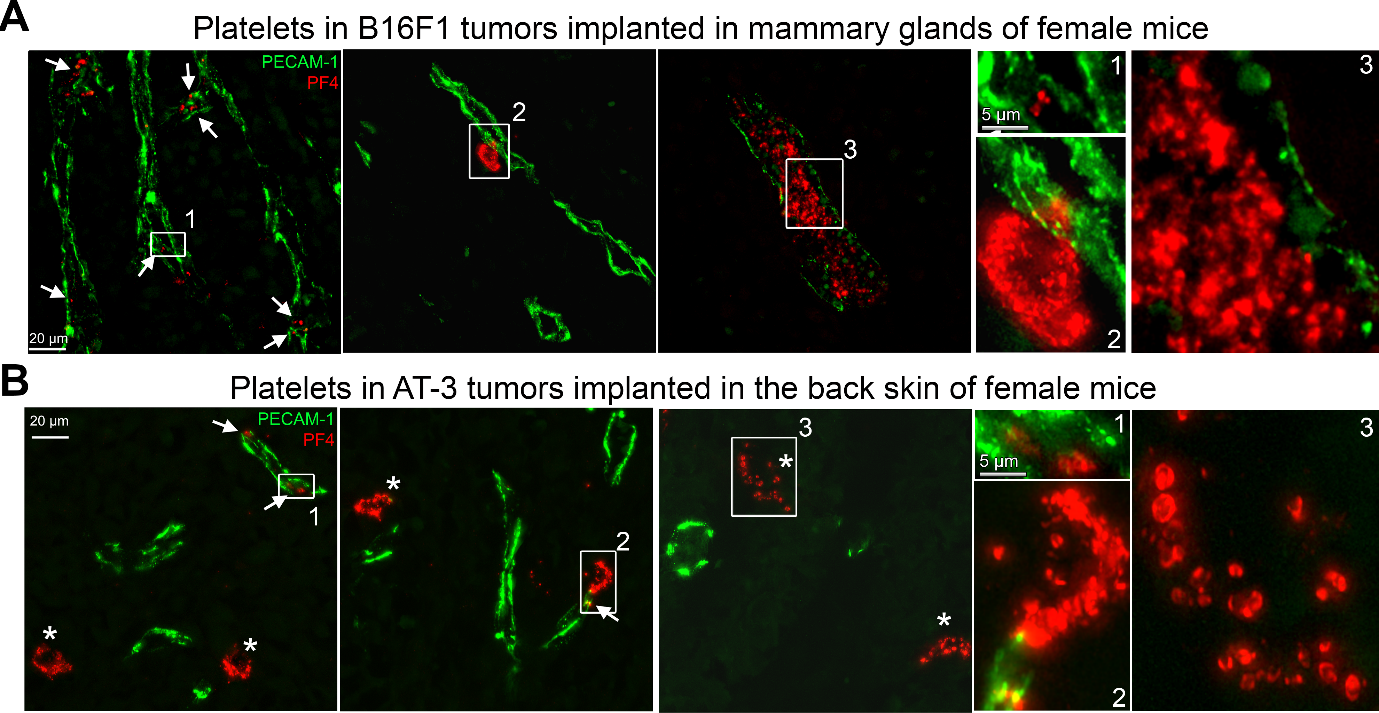


**Supplementary Figure 5. Localization of platelets in mammary B16F1 tumors and AT-3 back skin tumors. A.** Representative images of platelet factor 4 (PF4) and PECAM-1 staining in B16F1 tumors grown in mammary fat pads of female mice showing the presence of intravascular individual platelets (arrows, left panel), perivascular platelets (middle panel), and occlusive thrombi (right panel). Higher magnification views of the squared areas are shown on the right. **B.** Representative images of platelet factor 4 (PF4) and PECAM-1 staining in AT-3 tumors grown in the dorsal skin of female mice showing the presence of intravascular individual platelets (arrows, left and middle panels), perivascular platelets (middle panel), and of extravascular platelet clusters (asterisks, left middle and right panel). Higher magnification views of the squared areas are shown on the right.

**
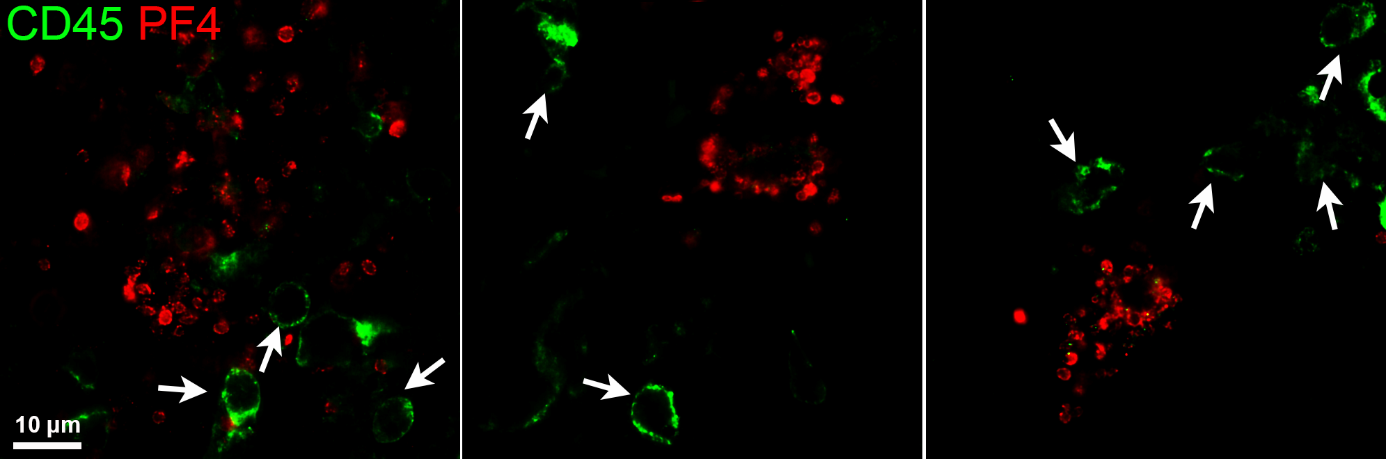
**

**Supplementary Figure 6. Extravascular platelet clusters are not associated with leukocyte infiltration in AT-3 tumors.** Representative images of CD45 and PF4 staining showing extravascular PF4-positive platelet clusters distinct from CD45-positive leukocytes (arrows) in mammary AT-3 tumors.


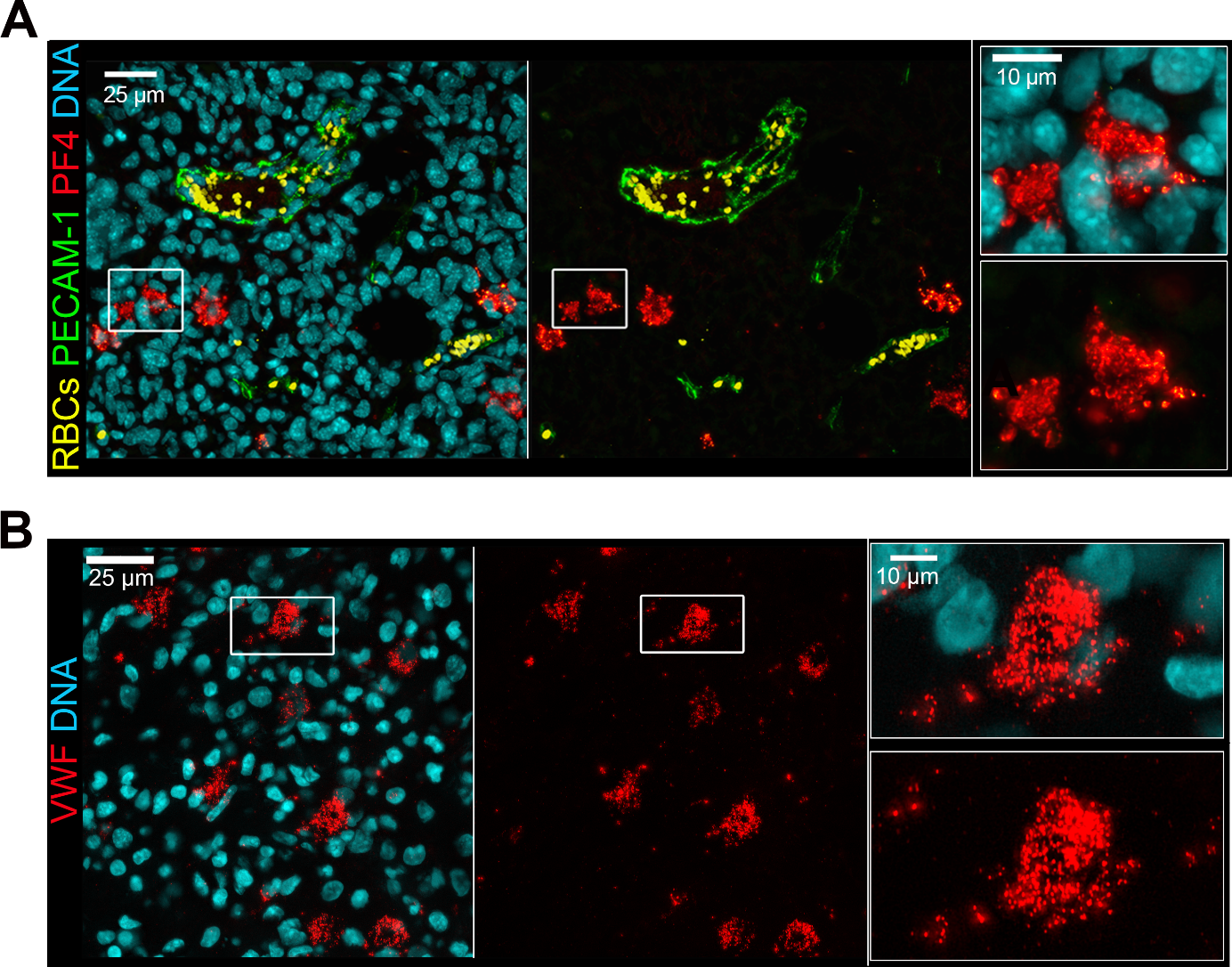


**Supplementary Figure 7. Extravascular platelet clusters in the microenvironment of AT-3 mammary tumors. A.** Representative images of PF4 and PECAM-1 staining in AT-3 tumors showing the presence of extravascular PF4-positive platelet clusters. Higher magnification views of the squared areas are shown on the right. **B.** Representative images of VWF staining in AT-3 tumors showing the presence of extravascular VWF-positive platelet clusters. Higher magnification views of the squared areas are shown on the right.

**
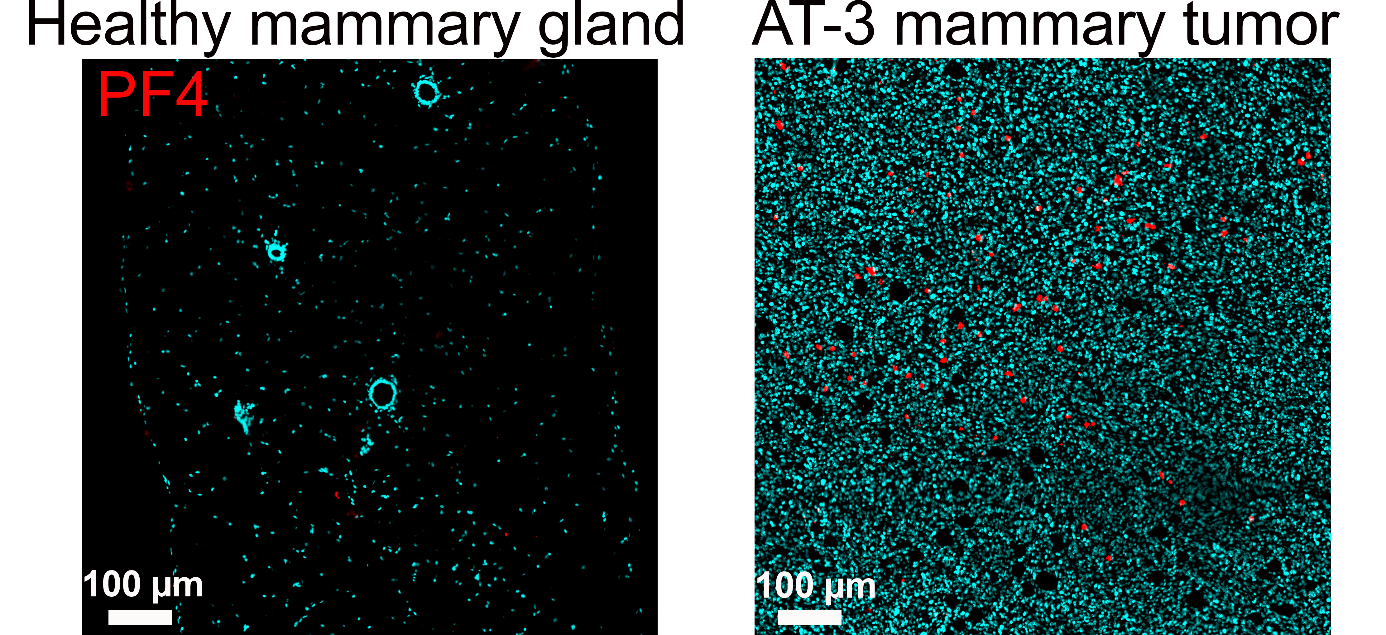
**

**Supplementary Figure 8. Extravascular PF4-positive platelet clusters are absent from healthy mammary glands.** Representative images of immunostaining for platelet factor 4 (PF4) in healthy mammary glands and AT-3 mammary tumors from wild-type mice showing an abundance of extravascular PF4-positive platelets in AT-3 mammary tumor.

**
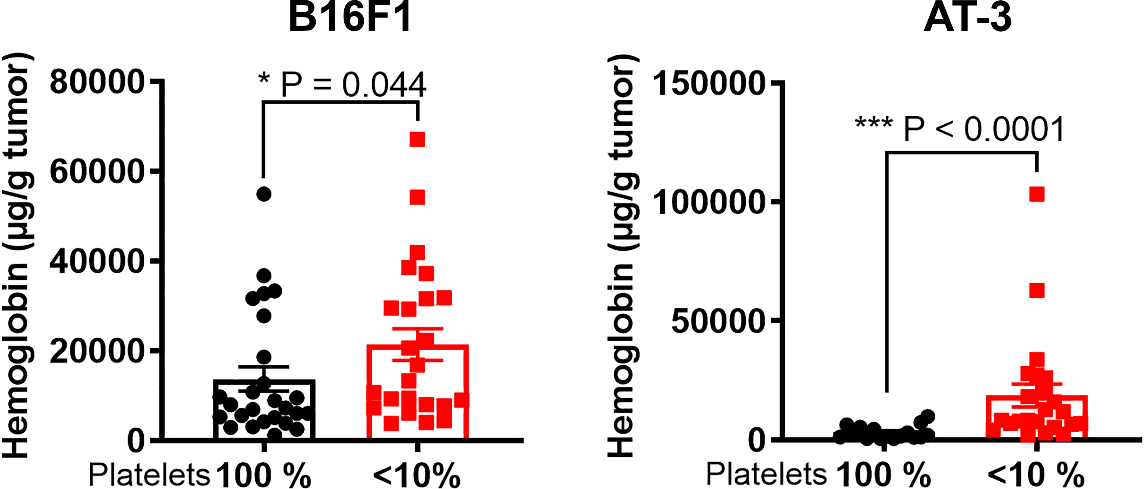
**

**Supplementary Figure 9. Chronic and severe thrombocytopenia increases tumor bleeding in both B16F1 and AT-3 tumors.** Comparison of hemoglobin content in B16F1 and AT-3 tumors from control mice and mice with chronic severe thrombocytopenia. n=26 (100%) and 24 (<10%) B16F1 tumors; n=16 (100%) and 23 (<10%) AT-3 tumors.

**
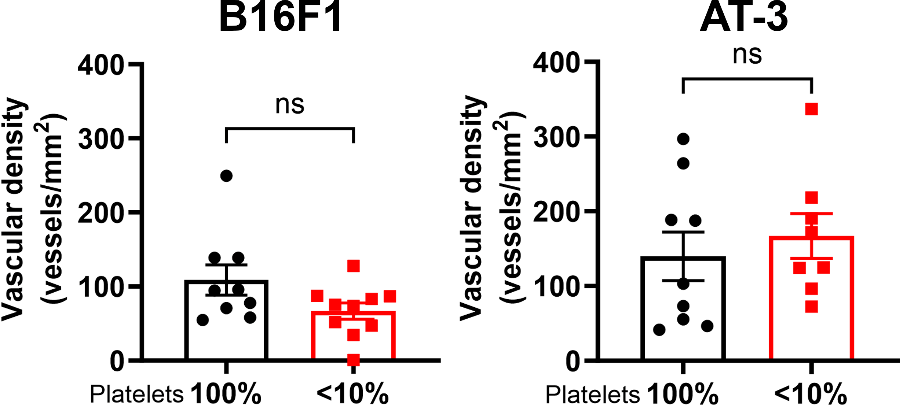
**

**Supplementary Figure 10. Vessel density B16F1 and AT-3 tumors is not affected by chronic and severe thrombocytopenia.** Quantification of vascular density in B16F1 and AT-3 tumors from control mice and mice with chronic severe thrombocytopenia. n=9 (100%) and 10 (<10%) B16F1 tumors; n=9 (100%) and 8 (<10%) AT-3 tumors. ns: non-significant.

**
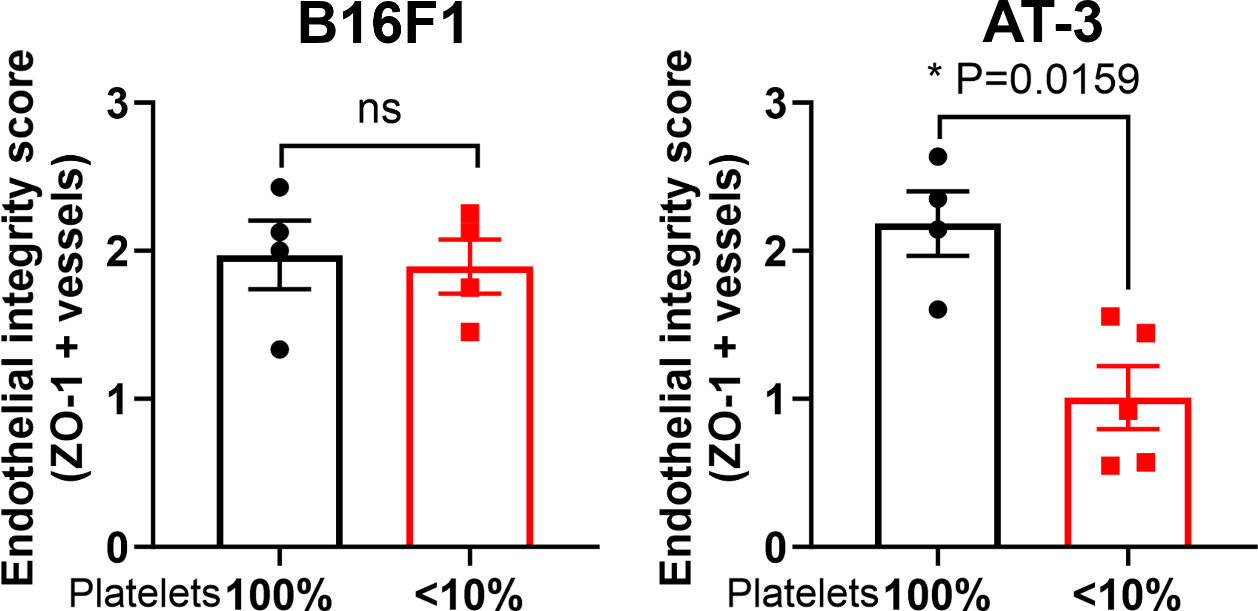
**

**Supplementary Figure 11. Impact of chronic and severe thrombocytopenia on endothelial integrity in B16F1 and AT-3 tumors.** Endothelial integrity scores of ZO-1 staining for evaluation of endothelial integrity in B16F1 and AT-3 tumors from control mice and mice with chronic severe thrombocytopenia. n=4 (100%) and 4 (<10%) B16F1 tumors; n=4 (100%) and 5 (<10%) AT-3 tumors. ns: non-significant.

**
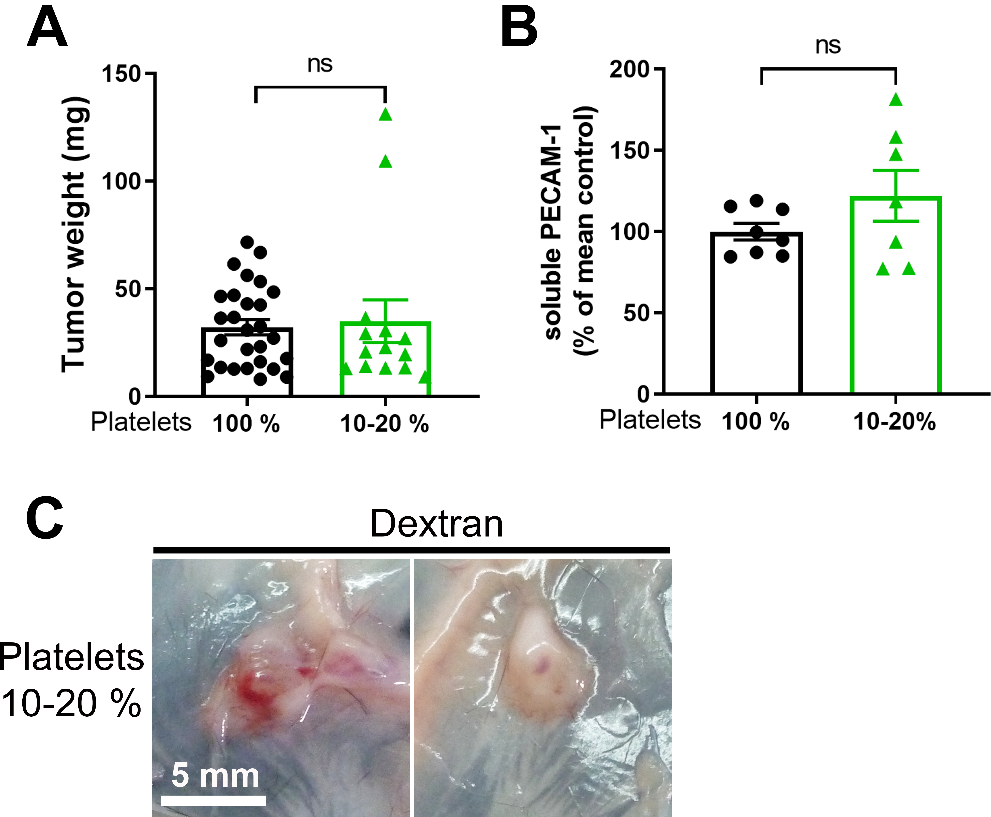
**

**Supplementary Figure 12. Tumor endothelial barrier function is maintained in c-mpl^-/-^ mice. A.** Wet weight of AT-3 tumors from c-mpl^+/+^ (platelet 100%, n=28) and c-mpl^-/-^ (platelets 10-20%, n=14). **B.** Comparison of soluble PECAM-1 levels in plasma from control c-mpl^+/+^ (platelets 100%, n=8) and thrombocytopenic c-mpl^-/-^ (platelets 10-20%, n=7) mice with AT-3. ns: non-significant. **C.** Representative images showing the macroscopic aspects of AT-3 tumors from thrombocytopenic c-mpl^-/-^ mice having received an intravenous injection of FITC-dextran (2000kDa) prior to sacrifice.

**
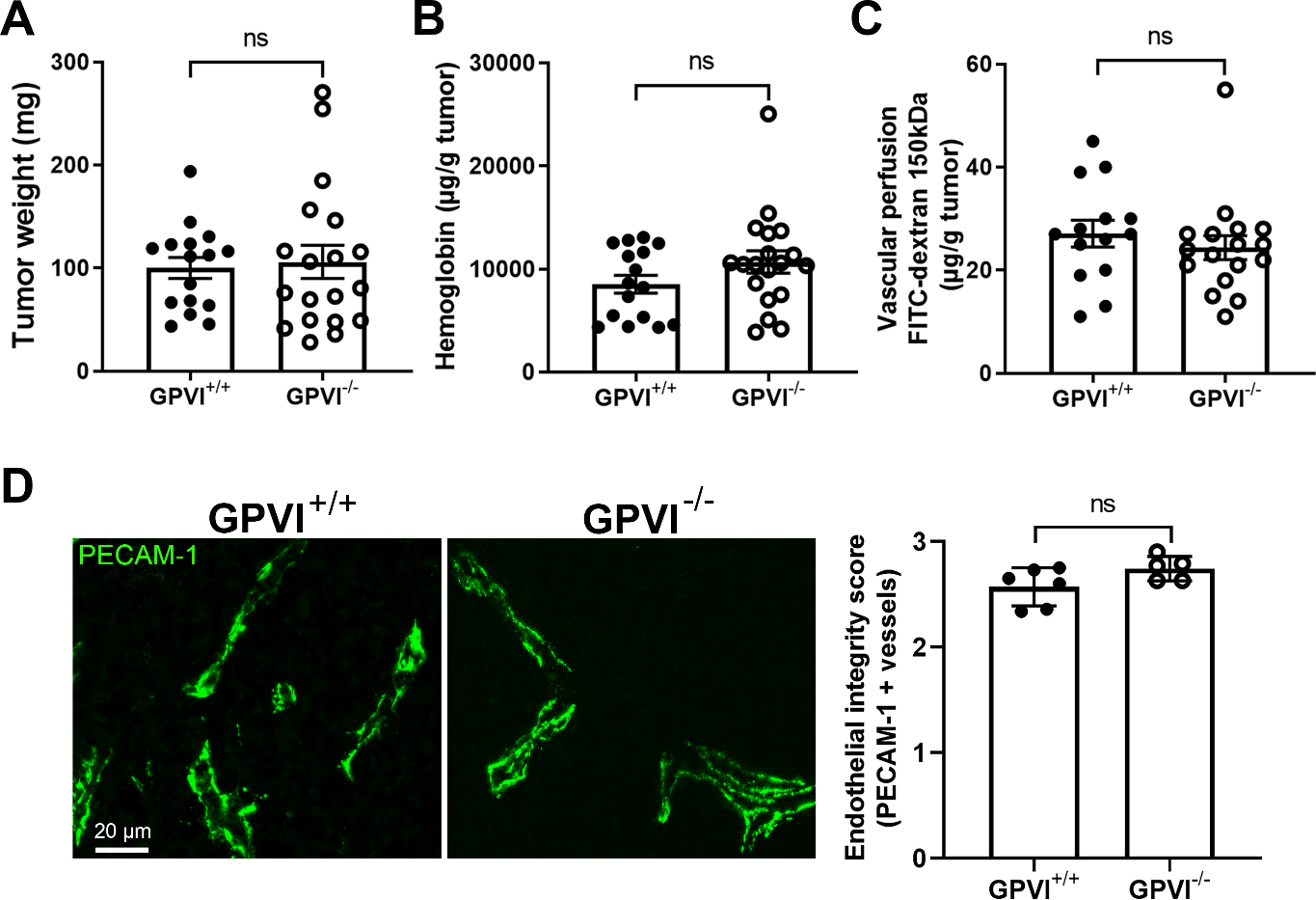
**

**Supplementary Figure 13. GPVI deficiency does not alter vascular permeability or integrity in AT-3 mammary tumors. A.** Comparison of wet weight of AT-3 tumors from GPVI+/+ and GPVI-/- mice. n=16 GPVI+/+ and 19 GPVI-/- tumors. **B.** Comparison of hemoglobin content in AT-3 tumors from GPVI+/+ and GPVI-/- mice. n=16 GPVI+/+ and 19 GPVI-/- tumors. **C.** Tumor vascular perfusion as assessed by measurement of intratumor FITC-dextran content of AT-3 tumors from GPVI+/+ and GPVI-/- mice explanted 5 min after intravenous injection of FITC-dextran, 150kDa. n=14 GPVI+/+ and 17 GPVI-/- tumors. **D.** Representative images and corresponding endothelial integrity scores of PECAM-1 staining for evaluation of endothelial integrity in AT-3 tumors from GPVI+/+ and GPVI-/- mice. n= 6 GPVI+/+ and 5 GPVI-/- tumors; each dot represents the mean score calculated from 20 to 30 vessels per tumor on random images taken with a 63x objective. ns: non significant.

**Supplemental Movie legends 1-10**

**Supplemental Movie 1-2-3**

Wild-type mice implanted with AT-3 tumors were injected intravenously with fluorochrome conjugated antibody to GPIX and FITC-dextran (2000 kDa) and were observed through a dorsal skinfold chamber at different magnifications.

**Supplemental Movie 4**

Wild-type mice implanted with AT-3 tumors were injected intravenously with fluorochrome conjugated antibody to GPIX, Gr-1 and PECAM-1. Platelet-tumor vessel interactions were observed in angiogenic sprouts.

**Supplemental Movie 5-6**

Wild-type mice implanted with B16F1 tumors were injected intravenously with fluorochrome conjugated antibody to GPIX and FITC-dextran (2000 kDa) and were observed through a dorsal skinfold chamber at different magnifications.

**Supplemental Movie 7**

Wild-type mice implanted with B16F1 tumors were injected intravenously with fluorochrome conjugated antibody to GPIX and Gr-1.

**Supplemental Movie 8**

Non-tumor bearing mice were injected intravenously with fluorochrome conjugated antibody to GPIX and FITC-dextran (2000 kDa) and were observed through a dorsal skinfold chamber.

**Supplemental Movie 9**

Wild-type mice implanted with AT-3 tumors were injected intravenously with fluorochrome conjugated antibody to GPIX and fibrin. Platelets associated with fibrin deposit were observed in the microcirculation.

**Supplemental Movie 10**

Wild-type mice implanted with B16F1 tumors were injected intravenously with fluorochrome conjugated antibody to GPIX and Gr-1. Platelets in interaction with migrating neutrophils were observed (white arrow). Occlusive microthrombi were observed in the tumor vasculature.
